# Supplementary material for: Akt phosphorylates insulin receptor substrate to limit PI3K-mediated PIP3 synthesis
Source: eLife. 2021 Jul 13;10:e66942. doi: 10.7554/eLife.66942 (PMC8277355; doi:10.7554/eLife.66942)
Supplement: Supplementary file 1. [file elife-66942-supp1.docx]

**Supplementary File 1a.** Reactions and rate equations for the PI3K/Akt pathway models.

| **Reaction Number** | | **Reaction** | | **Rate equation** | |
| --- | --- | --- | --- | --- | --- |
| R1 | | InsR → pInsR | | kf1 * InsR*(Ins0+InsB0)/(Km1 + InsR) - kr1*pInsR | |
| R4 | | PDPK1 + PIP3 → pmPDPK1 | | kf4*PDPK1*PIP3 - kr4 * pmPDPK1 | |
| R5 | | Akt + PIP3 → pmAkt | | kf5*Akt*PIP3 - kr5 * pmAkt | |
| R6 | | pmAkt → pmAkt309 | | kf6*pmAkt*pmPDPK1/ (Km6 +pmAkt) -kr6*pmAkt309 | |
| R7 | | pmAkt → pmAkt474 | | kf7 * pmAkt * amTorc2 / (Km7 + pmAkt) - kr7 * pmAkt474 | |
| R8 | | pmAkt309 → pmAkt309474 | | kf7 * pmAkt309 * amTorc2 / (Km8 + pmAkt309) - kr7 * pmAkt309474 | |
| R9 | | pmAkt474 → pmAkt309474 | | kf6 * pmAkt474 * pmPDPK1 / (Km9 + pmAkt474) - kr6 * pmAkt309474 | |
| R10 | | mTorc2 + PIP3 → pmTorc2 | | kf10*mTorc2*PIP3 - kr10 * pmTorc2 | |
| R11 | | mTorc2 → p_mTorc2 | | mTorc2*(kf11a*pmAkt309 + kf11b*pmAkt309474)/(Km11 + mTorc2) -kr11 * p_mTorc2 | |
| R12 | | mTorc1 → p_mTorc1 | | kf12 * mTorc1/(1+ki12*PRAS40) - kr12*p_mTorc1 | |
| R13 | | PRAS40 → p_PRAS40 | | PRAS40*(kf13a*pmAkt309 + kf13b*pmAkt309474)/(Km13 + PRAS40)-kr13*p_PRAS40 | |
| **Variation in the model structure** | | | | | |
| **Model** | **Reaction Number** | | **Reaction** | | **Rate equation** |
| 1 | R2 | | IRS → pIRS | | kf2*IRS*pInsR/((Km2 + IRS)*(1+ ki2a*p_mTorc1)) - kr2*pIRS |
|  | R3 | | PIP2 → PIP3 | | kf3*PIP2*pIRS/(Km3 + PIP2) - kr3*PIP3 |
| 2 | R2 | | IRS → pIRS | | kf2*IRS*pInsR/((Km2 + IRS)*(1+ ki2a*p_mTorc1)) - kr2*pIRS |
|  | R3 | | PIP2 → PIP3 | | kf3*PIP2*pIRS/(Km3 + PIP2) - kr3*PIP3 |
| 3 | R2 | | IRS → pIRS | | kf2*IRS*pInsR/(Km2 + IRS) - kr2*pIRS |
|  | R3 | | PIP2 → PIP3 | | kf3*PIP2*pIRS/(Km3 + PIP2) - kr3*PIP3 |
| 4 | R2 | | IRS → pIRS | | kf2*IRS*pInsR/((Km2 + IRS)*(1+ ki2a* pmPDPK1)) - kr2*pIRS |
|  | R3 | | PIP2 → PIP3 | | kf3*PIP2*pIRS/(Km3 + PIP2) - kr3*PIP3 |
| 5 | R2 | | IRS → pIRS | | kf2*IRS*pInsR/(Km2 + IRS) - kr2*pIRS |
|  | R3 | | PIP2 → PIP3 | | kf3*PIP2*pIRS/(Km3 + PIP2) - kr3*PIP3*(1+ ki2a* pmPDPK1) |
| 6 | R2 | | IRS → pIRS | | kf2*IRS*pInsR/((Km2 + IRS)*(1+ ki2a* (pmTorc2+ p_mTorc2))) - kr2*pIRS |
|  | R3 | | PIP2 → PIP3 | | kf3*PIP2*pIRS/(Km3 + PIP2) - kr3*PIP3 |
| 7 | R2 | | IRS → pIRS | | kf2*IRS*pInsR/(Km2 + IRS) - kr2*pIRS |
|  | R3 | | PIP2 → PIP3 | | kf3*PIP2*pIRS/(Km3 + PIP2) - kr3*PIP3*(1+ ki2a* (pmTorc2+ p_mTorc2)) |
| 8 | R2 | | IRS → pIRS | | kf2*IRS*pInsR/(Km2 + IRS) - kr2*pIRS |
|  | R3 | | PIP2 → PIP3 | | kf3*PIP2*pIRS/(Km3 + PIP2) - kr3*PIP3*(1+ ki2a* pmAkt309 + ki2b*pmAkt309474) |
| 9 | R2 | | IRS → pIRS | | kf2*IRS*pInsR/(Km2 + IRS) *(1+ ki2a* pmAkt309 + ki2b*pmAkt309474) - kr2*pIRS |
|  | R3 | | PIP2 → PIP3 | | kf3*PIP2*pIRS/(Km3 + PIP2) - kr3*PIP3 |

**Supplementary File 1b.** Ordinary differential equations of the PI3K/Akt pathway models. The reaction rates are given in Table S1. The initial conditions of the ODEs were taken from 3T3-L1 adipocyte iBAQ data [(Humphrey et al., 2013)](https://paperpile.com/c/hUzLJ3/jVNt).

| **Left hand Side** | **Right hand Side** | **Initial Conditions (nM)** | | | | | | | | |
| --- | --- | --- | --- | --- | --- | --- | --- | --- | --- | --- |
|  |  | **Model 1** | **Model 2** | **Model 3** | **Model 4** | **Model 5** | **Model 6** | **Model 7** | **Model 8** | **Model 9** |
| d[InsR]/dt | -R1 | 3.59E+00 | 3.59E+00 | 3.59E+00 | 3.59E+00 | 3.59E+00 | 3.58E+00 | 3.59E+00 | 3.59E+00 | 3.59E+00 |
| d[pInsR]/dt | +R1 | 1.26E-03 | 3.28E-03 | 4.84E-03 | 4.30E-03 | 2.50E-03 | 9.97E-03 | 3.66E-03 | 3.40E-03 | 1.38E-03 |
| d[IRS]/dt | -R2 | 3.59E+00 | 3.59E+00 | 3.59E+00 | 3.59E+00 | 3.59E+00 | 3.59E+00 | 3.59E+00 | 3.59E+00 | 3.57E+00 |
| d[pIRS]/dt | +R2 | 8.24E-03 | 1.57E-03 | 2.11E-03 | 2.31E-03 | 2.78E-03 | 9.25E-04 | 3.83E-04 | 4.97E-04 | 2.50E-02 |
| d[PIP2]/dt | -R3 | 1.45E+03 | 1.38E+03 | 1.45E+03 | 1.45E+03 | 1.45E+03 | 1.45E+03 | 1.45E+03 | 1.44E+03 | 1.45E+03 |
| d[PIP3]/dt | +R3-R4-R5-R10 | 1.42E-01 | 9.81E-01 | 4.12E-05 | 2.85E-01 | 3.97E-03 | 2.86E-02 | 1.30E-01 | 5.61E+00 | 2.95E-02 |
| d[PDPK1]/dt | -R4 | 2.76E+00 | 2.77E+00 | 2.77E+00 | 2.12E+00 | 2.48E+00 | 2.67E+00 | 2.32E+00 | 4.98E-02 | 8.82E-01 |
| d[pmPDPK1]/dt | R4 | 9.14E-03 | 4.90E-03 | 3.45E-03 | 6.53E-01 | 2.88E-01 | 1.01E-01 | 4.51E-01 | 2.72E+00 | 1.89E+00 |
| d[Akt]/dt | -R5 | 6.07E+02 | 5.36E+02 | 6.07E+02 | 6.06E+02 | 6.03E+02 | 6.07E+02 | 6.05E+02 | 6.07E+02 | 6.07E+02 |
| d[pmAkt]/dt | +R5-R6-R7 | 1.30E-01 | 1.39E+00 | 1.87E-01 | 5.21E-02 | 3.98E+00 | 3.67E-02 | 1.81E+00 | 4.18E-02 | 2.88E-01 |
| d[pmAkt309]/dt | +R6-R8 | 9.00E-05 | 1.00E-07 | 3.74E-02 | 2.64E-01 | 1.34E-02 | 2.76E-07 | 4.00E-01 | 3.12E-01 | 4.10E-01 |
| d[pmAkt474]/dt | +R7-R9 | 9.80E-04 | 7.02E+01 | 5.36E-06 | 5.82E-01 | 6.37E-07 | 4.28E-07 | 2.24E-05 | 2.93E-08 | 8.93E-08 |
| d[pmAkt309474]/dt | +R8+R9 | 2.55E-06 | 4.61E-05 | 3.59E-05 | 5.25E-03 | 5.65E-06 | 7.90E-11 | 5.97E-06 | 1.34E-04 | 5.34E-05 |
| d[PRAS40]/dt | -R13 | 2.89E+00 | 2.91E+00 | 3.03E+00 | 3.02E+00 | 2.87E+00 | 3.01E+00 | 2.88E+00 | 3.00E+00 | 2.95E+00 |
| d[p_PRAS40]/dt | R13 | 1.43E-01 | 1.24E-01 | 4.87E-03 | 5.35E-03 | 1.56E-01 | 2.18E-02 | 1.46E-01 | 3.41E-02 | 7.67E-02 |
| d[mTorc1]/dt | -R12 | 9.25E+00 | 7.40E-05 | 4.56E-02 | 9.25E+00 | 9.25E+00 | 9.25E+00 | 9.25E+00 | 9.25E+00 | 9.25E+00 |
| d[p_mTorc1]/dt | +R12 | 2.46E-01 | 9.50E+00 | 9.45E+00 | 2.46E-01 | 2.46E-01 | 2.46E-01 | 2.46E-01 | 2.46E-01 | 2.46E-01 |
| d[mTorc2]/dt | -R10-R11 | 3.30E-01 | 3.23E-01 | 3.30E-01 | 1.77E-01 | 3.29E-01 | 3.29E-01 | 2.85E-01 | 3.26E-01 | 2.63E-01 |
| d[p_mTorc2]/dt | R11 | 4.07E-04 | 2.33E-10 | 3.34E-04 | 1.19E-06 | 3.82E-04 | 1.67E-07 | 4.38E-02 | 3.62E-03 | 1.03E-02 |
| d[pmTorc2]/dt | R10 | 8.93E-06 | 7.40E-03 | 1.13E-08 | 1.53E-01 | 8.69E-04 | 1.43E-03 | 7.86E-04 | 4.10E-04 | 5.63E-02 |

**Supplementary File 1c.** Nine independent best-fitted parameter sets used for simulations.

| **Parameter** | **Model 1** | **Model 2** | **Model 3** | **Model 4** | **Model 5** | **Model 6** | **Model 7** | **Model 8** | **Model 9** | **Unit** |
| --- | --- | --- | --- | --- | --- | --- | --- | --- | --- | --- |
| Kf1 | 8.07E+03 | 4.38E+00 | 3.881504 | 4.06E+03 | 9.08E+03 | 1.73E-01 | 3.33E+02 | 3.59E+02 | 2.88E+02 | sec^-1^ |
| Km1 | 3.56E+01 | 1.59E+00 | 0.000998 | 3.76E-01 | 1.32E+01 | 3.42E-02 | 2.64E-01 | 1.61E+00 | 5.42E+00 | nM |
| Kr1 | 5.87E+03 | 9.25E+00 | 9.772372 | 8.55E+03 | 7.78E+03 | 1.72E-01 | 8.49E+02 | 7.28E+02 | 8.32E+02 | sec^-1^ |
| Kf2 | 5.00E-01 | 1.47E+01 | 0.004246 | 1.05E-02 | 1.16E+00 | 4.51E+00 | 1.57E+02 | 3.15E+00 | 7.38E-01 | sec^-1^ |
| Km2 | 3.31E+00 | 1.25E+00 | 0.134896 | 9.40E-03 | 3.08E-01 | 1.84E+00 | 7.93E-01 | 6.21E-02 | 4.06E-01 | nM |
| Ki2a | 6.64E-01 | 1.97E+01 | - | 1.45E-01 | 9.82E+01 | 7.16E+00 | 8.15E+01 | 2.10E-03 | 1.23E-01 | nM^-1^ |
| Ki2b | - | - | - | - | - | - | - | 3.77E+01 | 6.28E+02 | nM^-1^ |
| Kr2 | 3.42E-02 | 1.22E-01 | 0.027164 | 2.13E-02 | 9.62E-01 | 3.25E+01 | 1.23E+03 | 2.12E+01 | 3.38E-02 | sec^-1^ |
| Kf3 | 2.44E+00 | 5.58E+03 | 80.35261 | 9.93E+03 | 5.28E+00 | 2.04E+01 | 3.61E+01 | 1.22E+02 | 8.43E-01 | sec^-1^ |
| Km3 | 7.12E-02 | 8.71E+01 | 190.9853 | 1.64E+00 | 7.21E+01 | 1.55E+02 | 1.00E-04 | 6.38E+01 | 3.49E-01 | nM |
| Kr3 | 1.41E-01 | 8.39E+00 | 0.260615 | 8.05E+01 | 1.21E-01 | 5.96E-01 | 2.30E-02 | 1.03E-02 | 7.14E-01 | sec^-1^ |
| Kf4 | 7.08E+00 | 1.40E-02 | 78.88601 | 1.39E+02 | 7.98E-02 | 3.50E+03 | 5.73E+03 | 1.19E+01 | 5.56E+03 | nM^-1^ sec^-1^ |
| Kr4 | 3.04E+02 | 7.74E+00 | 1.393157 | 1.28E+02 | 2.70E-03 | 2.63E+03 | 3.83E+03 | 1.23E+00 | 7.66E+01 | sec^-1^ |
| Kf5 | 1.00E+00 | 7.57E-01 | 9.354057 | 1.21E+00 | 8.91E+03 | 1.06E+00 | 5.26E+01 | 3.00E-04 | 4.42E-01 | nM^-1^ sec^-1^ |
| Kr5 | 6.62E+02 | 2.86E+02 | 3396.253 | 4.01E+03 | 5.36E+03 | 5.00E+02 | 2.29E+03 | 2.24E+01 | 2.74E+01 | sec^-1^ |
| Kf6 | 5.10E-03 | 3.20E+01 | 0.508159 | 3.32E+03 | 9.93E+03 | 1.66E+00 | 6.59E+00 | 4.82E+01 | 3.62E+02 | sec^-1^ |
| Km6 | 2.02E-01 | 7.33E+02 | 104.472 | 1.76E+01 | 6.28E+03 | 7.85E+02 | 1.14E+02 | 3.27E+01 | 3.78E+01 | nM |
| Kr6 | 2.05E-01 | 2.97E+03 | 676.083 | 2.43E+01 | 1.36E+02 | 2.86E+01 | 1.16E-01 | 5.37E-01 | 1.26E+01 | sec^-1^ |
| Kf7 | 5.00E+00 | 1.85E+00 | 0.00032 | 3.20E+01 | 9.84E+00 | 1.35E-01 | 9.20E-03 | 8.10E-03 | 3.36E-03 | sec^-1^ |
| Km7 | 1.11E+01 | 3.69E-02 | 9462.372 | 2.97E+02 | 9.95E+03 | 2.36E+02 | 1.07E+03 | 4.25E+00 | 6.76E+02 | nM |
| Kr7 | 2.46E-02 | 2.00E-04 | 0.073114 | 3.80E-03 | 1.21E+03 | 7.03E-02 | 6.18E-02 | 3.30E-02 | 1.88E-02 | sec^-1^ |
| Km8 | 5.15E+01 | 8.09E+03 | 0.069024 | 9.31E+02 | 8.20E-03 | 7.08E+03 | 1.55E+02 | 1.93E+00 | 1.00E+02 | nM |
| Km9 | 7.81E-02 | 1.01E+01 | 668.3439 | 1.00E+04 | 5.92E+03 | 3.18E+01 | 8.43E+03 | 5.33E-02 | 9.08E-02 | nM |
| Kf10 | 1.96E-01 | 8.17E-02 | 6295.062 | 8.73E+03 | 1.90E-03 | 1.80E-03 | 7.71E+01 | 9.10E-03 | 8.85E+00 | nM^-1^ sec^-1^ |
| Kr10 | 1.03E+03 | 3.49E+00 | 8974.288 | 2.89E+03 | 2.90E-03 | 1.17E-02 | 3.64E+03 | 4.05E+02 | 1.22E+00 | sec^-1^ |
| Kf11a | 3.96E+03 | 5.06E+03 | 0.024889 | 3.58E-01 | 5.69E-01 | 4.76E-02 | 1.60E-01 | 3.76E-01 | 1.53E+02 | sec^-1^ |
| Kr11b | 4.60E+03 | 1.46E+02 | 0.000192 | 2.30E-03 | 3.24E-02 | 8.05E+03 | 6.75E+01 | 1.50E-02 | 8.43E+00 | sec^-1^ |
| Km11 | 8.71E-01 | 1.86E+03 | 5093.309 | 9.84E+03 | 1.14E+02 | 6.00E-04 | 5.68E+02 | 8.79E+02 | 4.51E-03 | nM |
| Kr11 | 2.48E+02 | 5.41E+03 | 2824.88 | 1.43E+00 | 5.71E-02 | 3.87E+00 | 7.00E-04 | 1.20E-02 | 6.03E+03 | sec^-1^ |
| Kf12 | 2.75E+00 | 8.20E+03 | 3741.106 | 2.75E+00 | 2.75E+00 | 2.75E+00 | 2.75E+00 | 2.75E+00 | 2.75E+00 | sec^-1^ |
| Ki12 | 1.90E+03 | 3.28E+01 | 0.554626 | 1.90E+03 | 1.90E+03 | 1.90E+03 | 1.90E+03 | 1.90E+03 | 1.90E+03 | nM^-1^ |
| Kr12 | 1.88E-02 | 7.00E-04 | 8.994976 | 1.88E-02 | 1.88E-02 | 1.88E-02 | 1.88E-02 | 1.88E-02 | 1.88E-02 | sec^-1^ |
| kf13a | 2.03E+03 | 3.33E-01 | 77.09035 | 3.84E+00 | 1.80E-03 | 9.25E+03 | 1.06E-02 | 1.80E-03 | 3.50E-02 | sec^-1^ |
| kf13b | 3.61E+01 | 2.67E+02 | 86.09938 | 1.27E+03 | 3.94E+02 | 5.37E-02 | 4.53E+01 | 4.44E+01 | 6.98E+03 | sec^-1^ |
| kr13 | 1.54E-03 | 1.80E-03 | 0.00228 | 3.27E+02 | 1.26E-02 | 3.00E-04 | 2.70E-03 | 7.94E-02 | 2.15E-02 | sec^-1^ |
| Km13 | 2.38E+03 | 1.54E+02 | 0.554626 | 1.03E+01 | 4.16E-01 | 1.37E+03 | 3.01E+01 | 4.16E+00 | 6.90E+02 | nM |
| InsB0 | 1.00E-02 | 1.00E-02 | 0.01 | 1.00E-02 | 1.00E-02 | 1.00E-02 | 1.00E-02 | 1.00E-02 | 1.00E-02 | nM |
| Ins0 | 1.00E+02 | 1.00E+02 | 100 | 1.00E+02 | 1.00E+02 | 1.00E+02 | 1.00E+02 | 1.00E+02 | 1.00E+02 | nM |
