# Supplementary material for: Akt phosphorylates insulin receptor substrate to limit PI3K-mediated PIP3 synthesis
Source: eLife. 2021 Jul 13;10:e66942. doi: 10.7554/eLife.66942 (PMC8277355; doi:10.7554/eLife.66942)
Supplement: Supplementary file 2. [file elife-66942-supp2.pdf]

Parameter identifiability analysis of Models 4 to 9

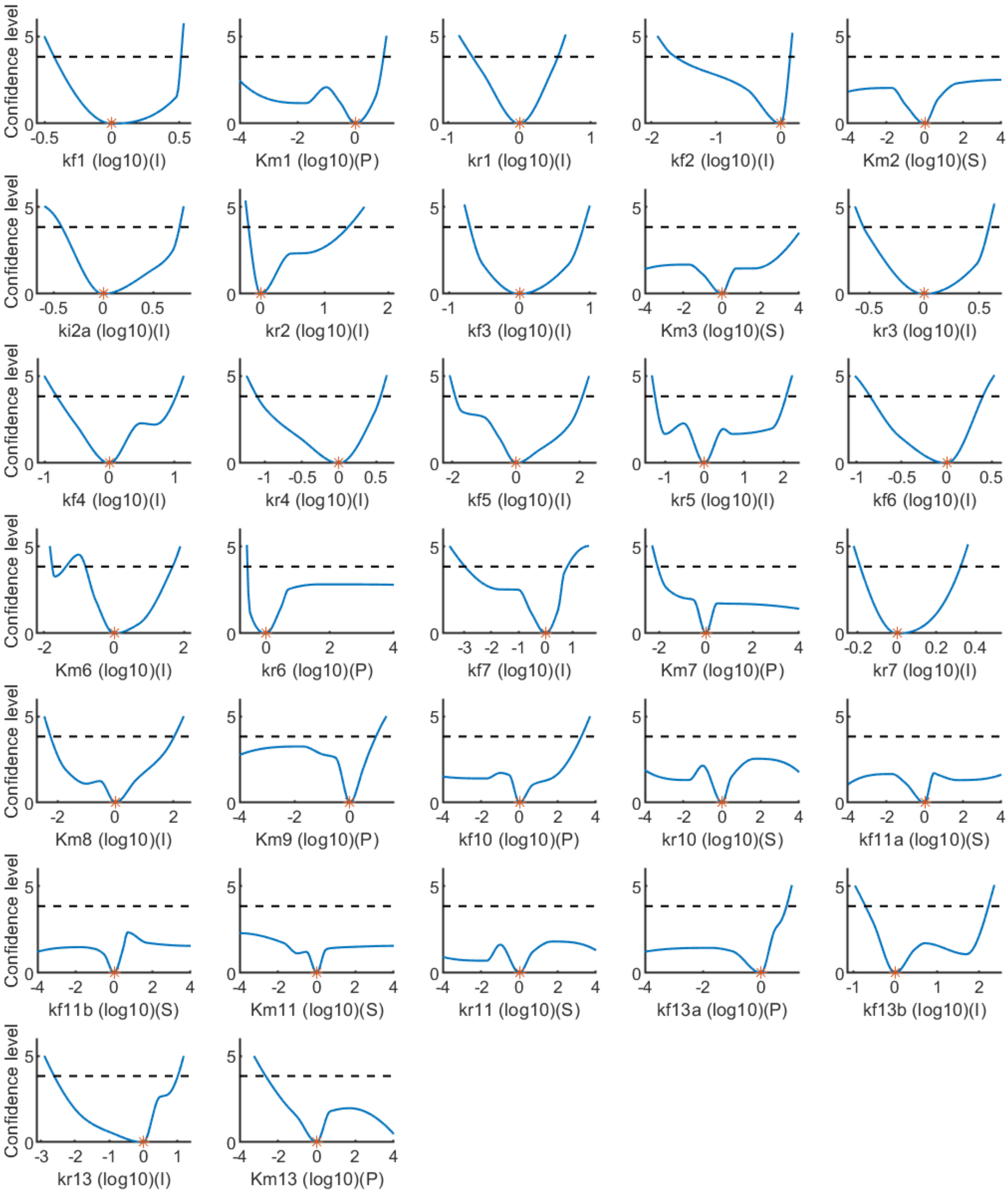

Figure 1. Parameter identifiability analysis of Model 4 (PDK1 to IRS/PI3K). The blue lines display profile likelihood versus model parameters, as computed in (1). For each parameter, the calibrated value is the lowest point within its corresponding blue curve (red star). X axis shows the relative value of the parameter compared to its calibrated value. The black dashed line indicates the threshold for 95% point-wise confidence interval. Parameter values are given in logarithm format. The type of identifiability of each parameter is given in the parenthesis (X-axis label) based on (1): I: Identifiable, P: practically non-identifiable, S: structurally non-identifiable. For more details please see Materials and Methods.

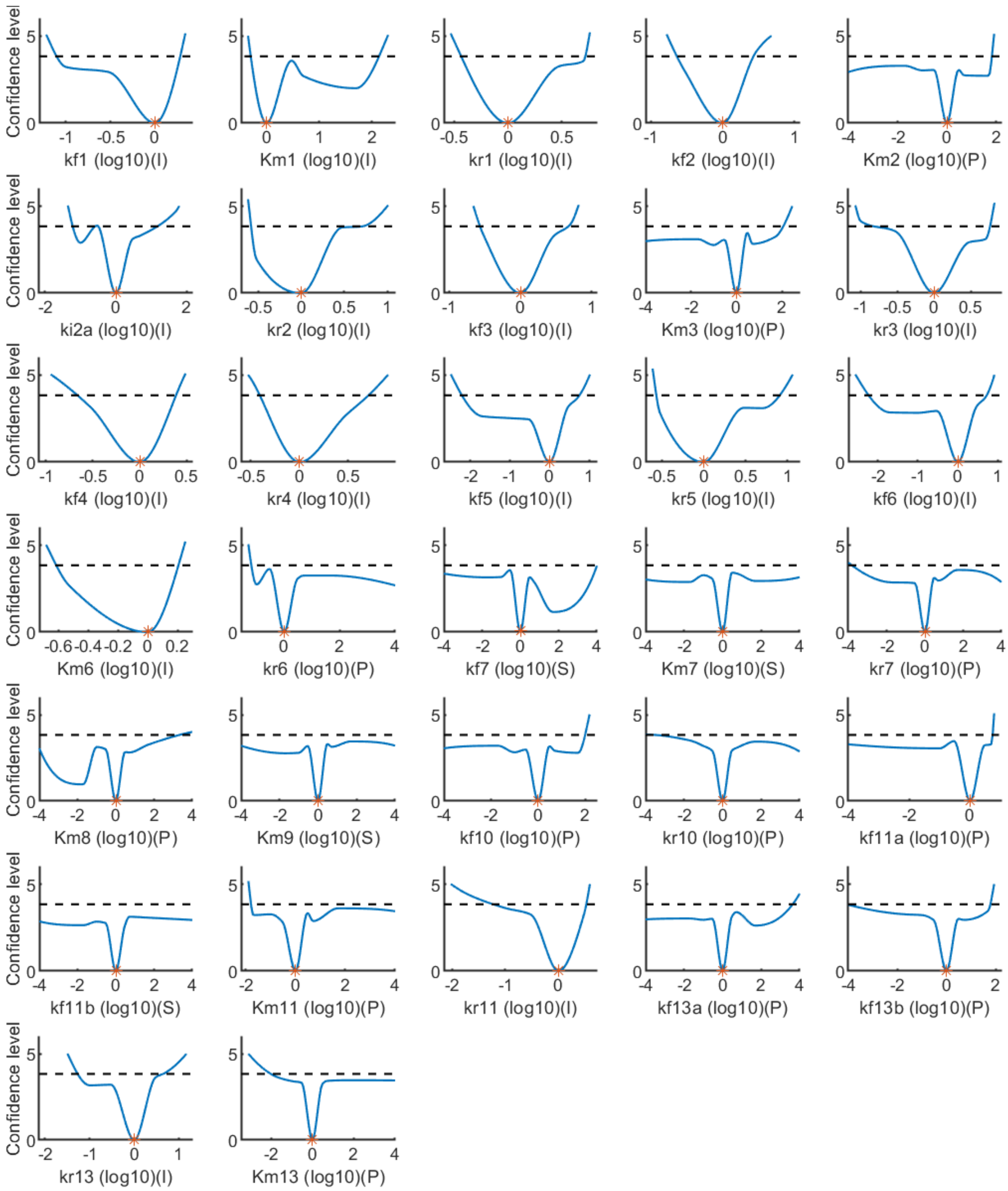

Figure 2. Parameter identifiability analysis of Model 5 (PDK1 to PTEN). The blue lines display profile likelihood versus model parameters, as computed in (1). For each parameter, the calibrated value is the lowest point within its corresponding blue curve (red star). X axis shows the relative value of the parameter compared to its calibrated value. The black dashed line indicates the threshold for 95% point-wise confidence interval. Parameter values are given in logarithm format. The type of identifiability of each parameter is given in the parenthesis (X-axis label) based on (1): I: Identifiable, P: practically non-identifiable, S: structurally non-identifiable. For more details please see Materials and Methods.

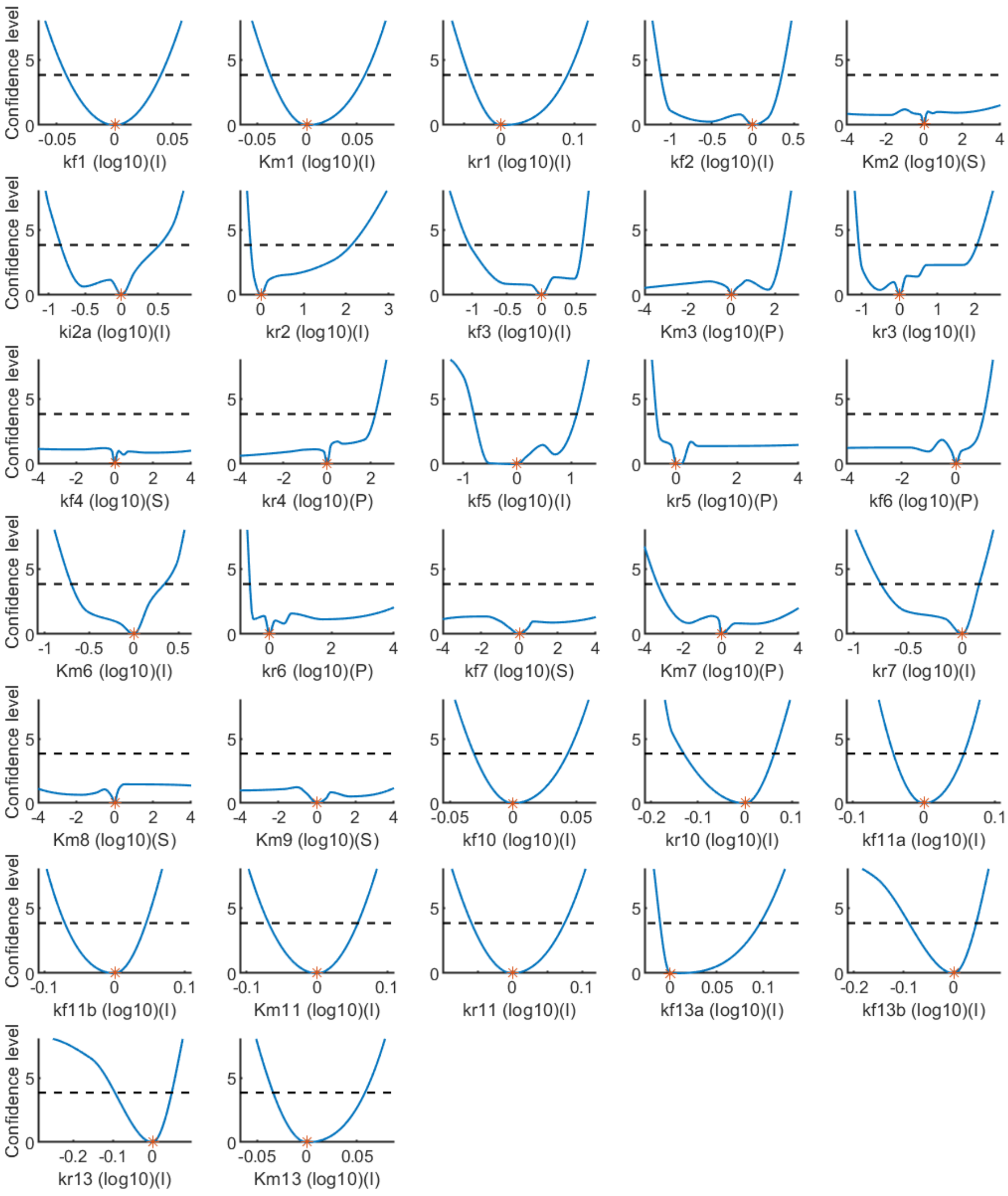

Figure 3. Parameter identifiability analysis of Model 6 (mTORC2 to IRS/PI3K). The blue lines display profile likelihood versus model parameters, as computed in (1). For each parameter, the calibrated value is the lowest point within its corresponding blue curve (red star). X axis shows the relative value of the parameter compared to its calibrated value. The black dashed line indicates the threshold for 95% point-wise confidence interval. Parameter values are given in logarithm format. The type of identifiability of each parameter is given in the parenthesis (X-axis label) based on (1): I: Identifiable, P: practically non-identifiable, S: structurally non-identifiable. For more details please see Materials and Methods.

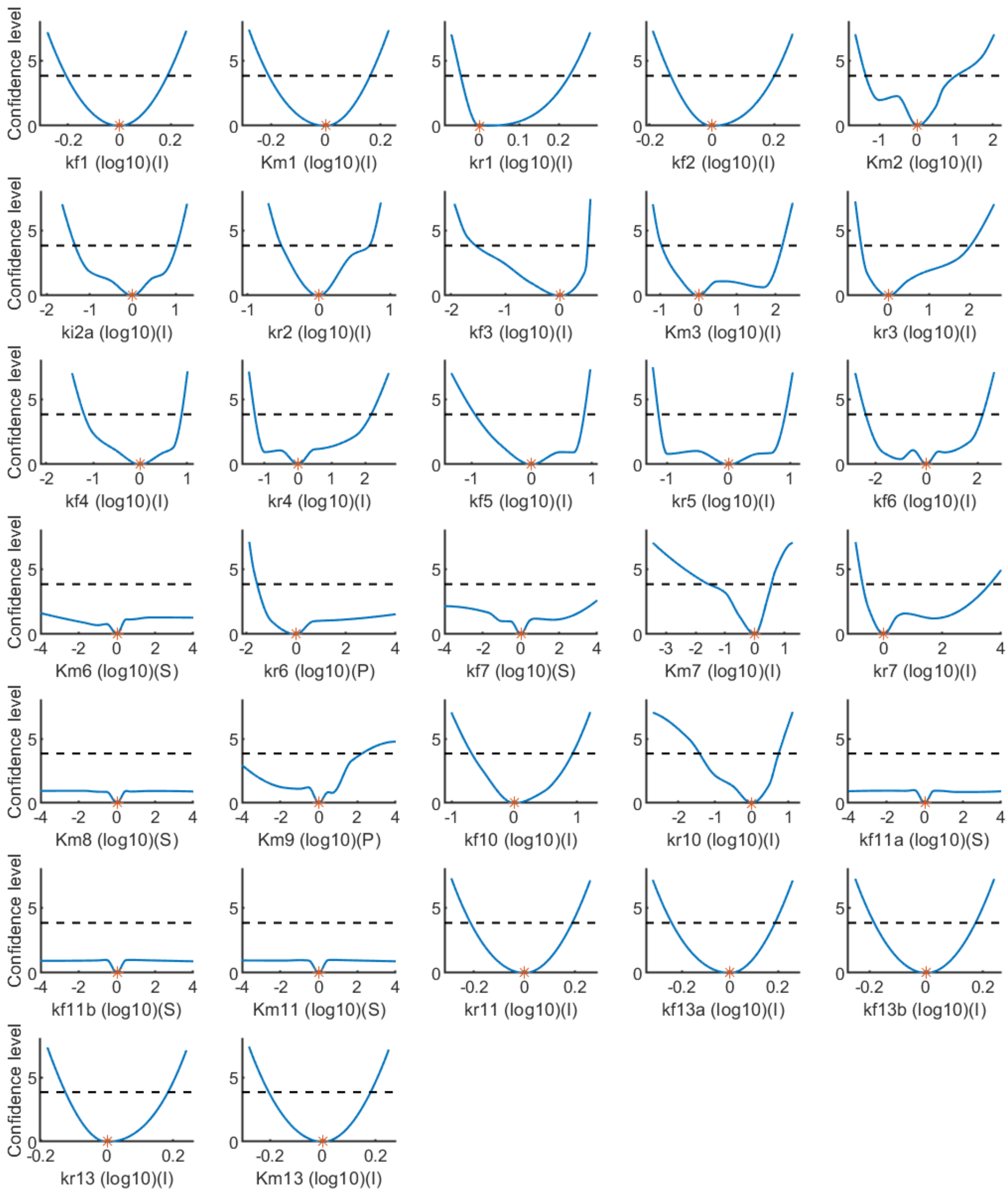

Figure 4. Parameter identifiability analysis of Model 7 (mTORC2 to PTEN). The blue lines display profile likelihood versus model parameters, as computed in (1). For each parameter, the calibrated value is the lowest point within its corresponding blue curve (red star). X axis shows the relative value of the parameter compared to its calibrated value. The black dashed line indicates the threshold for 95% point-wise confidence interval. Parameter values are given in logarithm format. The type of identifiability of each parameter is given in the parenthesis (X-axis label) based on (1): I: Identifiable, P: practically non-identifiable, S: structurally non-identifiable. For more details please see Materials and Methods.

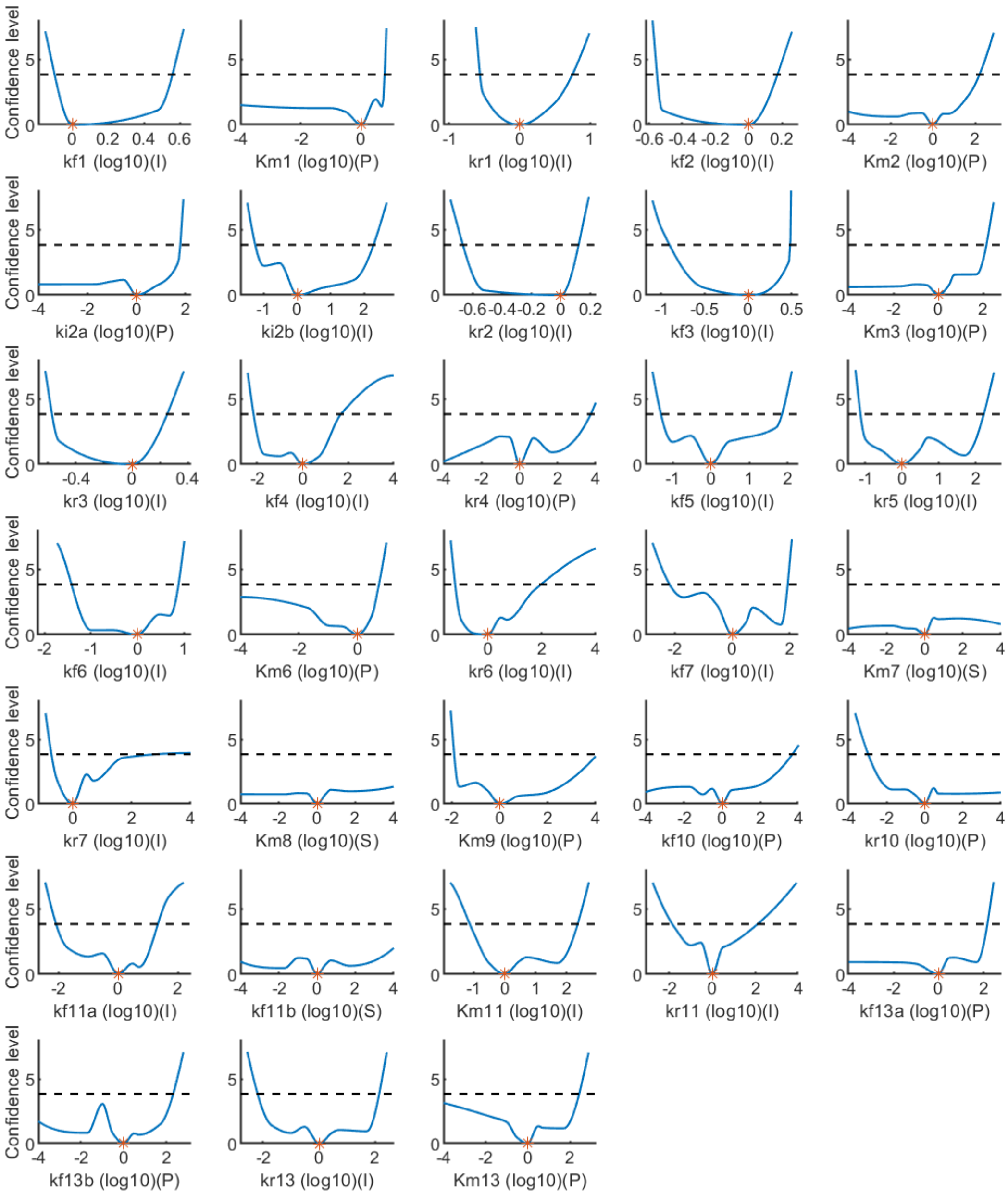

Figure 5. Parameter identifiability analysis of Model 8 (Akt to PTEN). The blue lines display profile likelihood versus model parameters, as computed in (1). For each parameter, the calibrated value is the lowest point within its corresponding blue curve (red star). X axis shows the relative value of the parameter compared to its calibrated value. The black dashed line indicates the threshold for 95% point-wise confidence interval. Parameter values are given in logarithm format. The type of identifiability of each parameter is given in the parenthesis (X-axis label) based on (1): I: Identifiable, P: practically non-identifiable, S: structurally non-identifiable. For more details please see Materials and Methods.

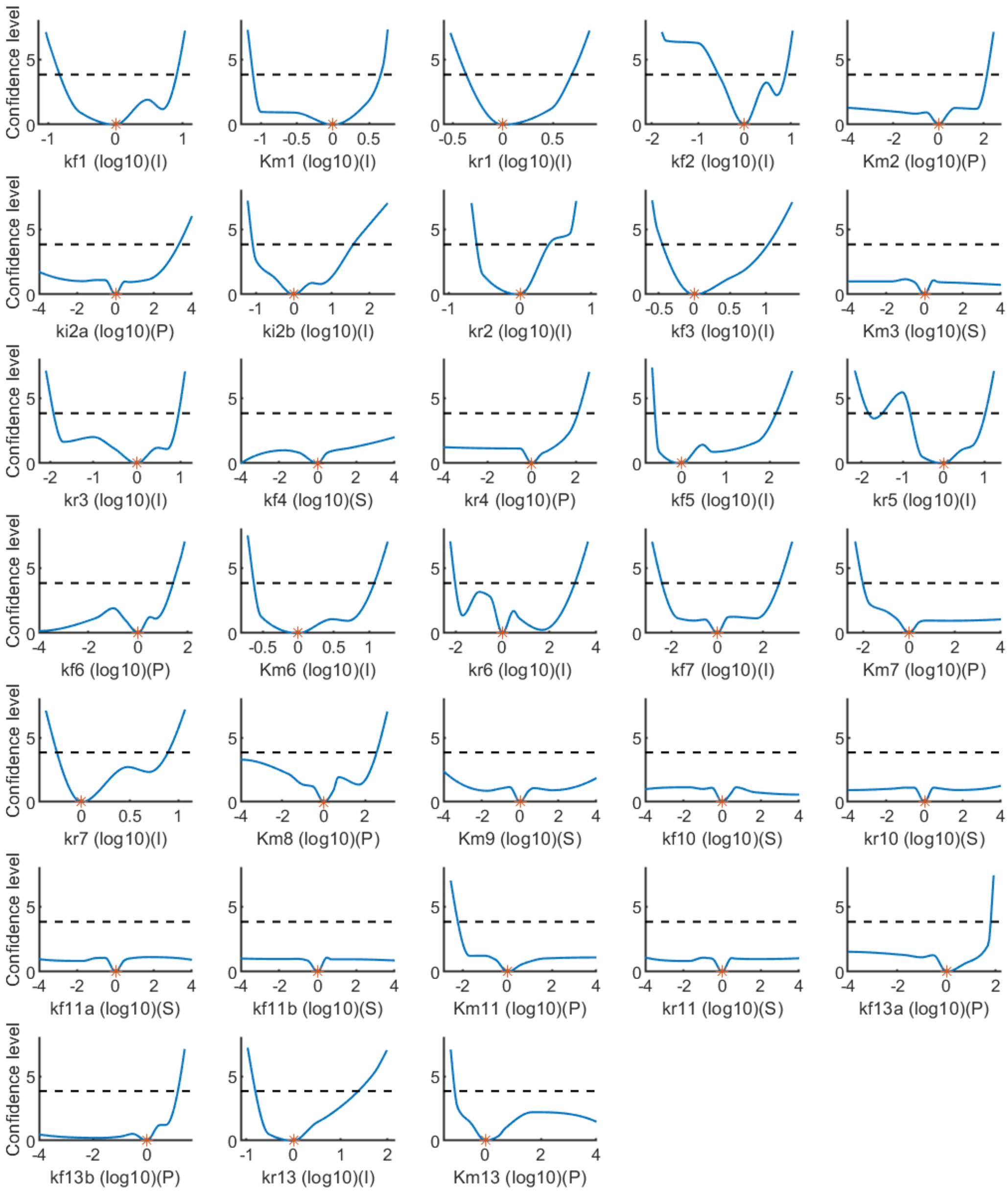

Figure 6. Parameter identifiability analysis of Model 9 (Akt to IRS/PI3K). The blue lines display profile likelihood versus model parameters, as computed in (1). For each parameter, the calibrated value is the lowest point within its corresponding blue curve (red star). X axis shows the relative value of the parameter compared to its calibrated value. The black dashed line indicates the threshold for 95% point-wise confidence interval. Parameter values are given in logarithm format. The type of identifiability of each parameter is given in the parenthesis (X-axis label) based on (1): I: Identifiable, P: practically non-identifiable, S: structurally non-identifiable. For more details please see Materials and Methods.

Reference:

(1) Raue A, Kreutz C, Maiwald T, Bachmann J, Schilling M, Klingmuller U, et al. Structural and practical identifiability analysis of partially observed dynamical models by exploiting the profile likelihood. Bioinformatics. 2009;25(15):1923-9.
