# Supplementary material for: Akt phosphorylates insulin receptor substrate to limit PI3K-mediated PIP3 synthesis
Source: eLife. 2021 Jul 13;10:e66942. doi: 10.7554/eLife.66942 (PMC8277355; doi:10.7554/eLife.66942)
Supplement: Supplementary file 3. [file elife-66942-supp3.docx]

**Supplementary File 3a.** DNA sequence of plasmids generated in this study.

| **Plasmid** | **DNA Sequence (5'-3')** |
| --- | --- |
| TagRFP-T-Akt2 | GACGGATCGGGAGATCTCCCGATCCCCTATGGTGCACTCTCAGTACAATCTGCTCTGATGCCGCATAGTTAAGCCAGTATCTGCTCCCTGCTTGTGTGTTGGAGGTCGCTGAGTAGTGCGCGAGCAAAATTTAAGCTACAACAAGGCAAGGCTTGACCGACAATTGCATGAAGAATCTGCTTAGGGTTAGGCGTTTTGCGCTGCTTCGCGATGTACGGGCCAGATATACGCGTTGACATTGATTATTGACTAGTTATTAATAGTAATCAATTACGGGGTCATTAGTTCATAGCCCATATATGGAGTTCCGCGTTACATAACTTACGGTAAATGGCCCGCCTGGCTGACCGCCCAACGACCCCCGCCCATTGACGTCAATAATGACGTATGTTCCCATAGTAACGCCAATAGGGACTTTCCATTGACGTCAATGGGTGGAGTATTTACGGTAAACTGCCCACTTGGCAGTACATCAAGTGTATCATATGCCAAGTACGCCCCCTATTGACGTCAATGACGGTAAATGGCCCGCCTGGCATTATGCCCAGTACATGACCTTATGGGACTTTCCTACTTGGCAGTACATCTACGTATTAGTCATCGCTATTACCATGGTGATGCGGTTTTGGCAGTACATCAATGGGCGTGGATAGCGGTTTGACTCACGGGGATTTCCAAGTCTCCACCCCATTGACGTCAATGGGAGTTTGTTTTGGCACCAAAATCAACGGGACTTTCCAAAATGTCGTAACAACTCCGCCCCATTGACGCAAATGGGCGGTAGGCGTGTACGGTGGGAGGTCTATATAAGCAGAGCTCTCTGGCTAACTAGAGAACCCACTGCTTACTGGCTTATCGAAATTAATACGACTCACTATAGGGAGACCCAAGCTGGCTAGACACCGCCACCATGGTGTCTAAGGGCGAAGAGCTGATTAAGGAGAACATGCACATGAAGCTGTACATGGAGGGCACCGTGAACAACCACCACTTCAAGTGCACATCCGAGGGCGAAGGCAAGCCCTACGAGGGCACCCAGACCATGAGAATCAAGGTGGTCGAGGGCGGCCCTCTCCCCTTCGCCTTCGACATCCTGGCTACCAGCTTCATGTACGGCAGCAGAACCTTCATCAACCACACCCAGGGCATCCCCGATTTCTTTAAGCAGTCCTTCCCTGAGGGCTTCACATGGGAGAGAGTCACCACATACGAAGACGGGGGCGTGCTGACCGCTACCCAGGACACCAGCCTCCAGGACGGCTGCCTCATCTACAACGTCAAGATCAGAGGGGTGAACTTCCCATCCAACGGCCCTGTGATGCAGAAGAAAACACTCGGCTGGGAGGCCAACACCGAGATGCTGTACCCCGCTGACGGCGGCCTGGAAGGCAGAACCGACATGGCCCTGAAGCTCGTGGGCGGGGGCCACCTGATCTGCAACTTCAAGACCACATACAGATCCAAGAAACCCGCTAAGAACCTCAAGATGCCCGGCGTCTACTATGTGGACCACAGACTGGAAAGAATCAAGGAGGCCGACAAAGAGACCTACGTCGAGCAGCACGAGGTGGCTGTGGCCAGATACTGCGACCTCCCTAGCAAACTGGGGCACAAACTTAATGGCATGGACGAGCTGTACAAGAATGAGGTGTCTGTCATCAAAGAAGGCTGGCTCCACAAGCGTGGTGAATACATCAAGACCTGGAGGCCACGGTACTTCCTGCTGAAGAGCGACGGCTCCTTCATTGGGTACAAGGAGAGGCCCGAGGCCCCTGATCAGACTCTACCCCCCTTAAACAACTTCTCCGTAGCAGAATGCCAGCTGATGAAGACCGAGAGGCCGCGACCCAACACCTTTGTCATACGCTGCCTGCAGTGGACCACAGTCATCGAGAGGACCTTCCACGTGGATTCTCCAGACGAGAGGGAGGAGTGGATGCGGGCCATCCAGATGGTCGCCAACAGCCTCAAGCAGCGGGCCCCAGGCGAGGACCCCATGGACTACAAGTGTGGCTCCCCCAGTGACTCCTCCACGACTGAGGAGATGGAAGTGGCGGTCAGCAAGGCACGGGCTAAAGTGACCATGAATGACTTCGACTATCTCAAACTCCTTGGCAAGGGAACCTTTGGCAAAGTCATCCTGGTGCGGGAGAAGGCCACTGGCCGCTACTACGCCATGAAGATCCTGCGGAAGGAAGTCATCATTGCCAAGGATGAAGTCGCTCACACAGTCACCGAGAGCCGGGTCCTCCAGAACACCAGGCACCCGTTCCTCACTGCGCTGAAGTATGCCTTCCAGACCCACGACCGCCTGTGCTTTGTGATGGAGTATGCCAACGGGGGTGAGCTGTTCTTCCACCTGTCCCGGGAGCGTGTCTTCACAGAGGAGCGGGCCCGGTTTTATGGTGCAGAGATTGTCTCGGCTCTTGAGTACTTGCACTCGCGGGACGTGGTATACCGCGACATCAAGCTGGAAAACCTCATGCTGGACAAAGATGGCCACATCAAGATCACTGACTTTGGCCTCTGCAAAGAGGGCATCAGTGACGGGGCCACCATGAAAACCTTCTGTGGGACCCCGGAGTACCTGGCGCCTGAGGTGCTGGAGGACAATGACTATGGCCGGGCCGTGGACTGGTGGGGGCTGGGTGTGGTCATGTACGAGATGATGTGCGGCCGCCTGCCCTTCTACAACCAGGACCACGAGCGCCTCTTCGAGCTCATCCTCATGGAAGAGATCCGCTTCCCGCGCACGCTCAGCCCCGAGGCCAAGTCCCTGCTTGCTGGGCTGCTTAAGAAGGACCCCAAGCAGAGGCTTGGTGGGGGGCCCAGCGATGCCAAGGAGGTCATGGAGCACAGGTTCTTCCTCAGCATCAACTGGCAGGACGTGGTCCAGAAGAAGCTCCTGCCACCCTTCAAACCTCAGGTCACGTCCGAGGTCGACACAAGGTACTTCGATGATGAATTTACCGCCCAGTCCATCACAATCACACCCCCTGACCGCTATGACAGCCTGGGCTTACTGGAGCTGGACCAGCGGACCCACTTCCCCCAGTTCTCCTACTCGGCCAGCATCCGCGAGTGAGACCCAGCTTTCTTGTACAAAGTGGTGATAATTAATTAAGATAAACCCGCTGATCAGCCTCGACTGTGCCTTCTAGTTGCCAGCCATCTGTTGTTTGCCCCTCCCCCGTGCCTTCCTTGACCCTGGAAGGTGCCACTCCCACTGTCCTTTCCTAATAAAATGAGGAAATTGCATCGCATTGTCTGAGTAGGTGTCATTCTATTCTGGGGGGTGGGGTGGGGCAGGACAGCAAGGGGGAGGATTGGGAAGACAATAGCAGGCATGCTGGGGATGCGGTGGGCTCTATGGCTTCTGAGGCGGAAAGAACCAGCTGGGGCTCTAGGGGGTATCCCCACGCGCCCTGTAGCGGCGCATTAAGCGCGGCGGGTGTGGTGGTTACGCGCAGCGTGACCGCTACACTTGCCAGCGCCCTAGCGCCCGCTCCTTTCGCTTTCTTCCCTTCCTTTCTCGCCACGTTCGCCGGCTTTCCCCGTCAAGCTCTAAATCGGGGGCTCCCTTTAGGGTTCCGATTTAGTGCTTTACGGCACCTCGACCCCAAAAAACTTGATTAGGGTGATGGTTCACGTAGTGGGCCATCGCCCTGATAGACGGTTTTTCGCCCTTTGACGTTGGAGTCCACGTTCTTTAATAGTGGACTCTTGTTCCAAACTGGAACAACACTCAACCCTATCTCGGTCTATTCTTTTGATTTATAAGGGATTTTGCCGATTTCGGCCTATTGGTTAAAAAATGAGCTGATTTAACAAAAATTTAACGCGAATTAATTCTGTGGAATGTGTGTCAGTTAGGGTGTGGAAAGTCCCCAGGCTCCCCAGCAGGCAGAAGTATGCAAAGCATGCATCTCAATTAGTCAGCAACCAGGTGTGGAAAGTCCCCAGGCTCCCCAGCAGGCAGAAGTATGCAAAGCATGCATCTCAATTAGTCAGCAACCATAGTCCCGCCCCTAACTCCGCCCATCCCGCCCCTAACTCCGCCCAGTTCCGCCCATTCTCCGCCCCATGGCTGACTAATTTTTTTTATTTATGCAGAGGCCGAGGCCGCCTCTGCCTCTGAGCTATTCCAGAAGTAGTGAGGAGGCTTTTTTGGAGGCCTAGGCTTTTGCAAAAAGCTCCCGGGAGCTTGTATATCCATTTTCGGATCTGATCAAGAGACAGGATGAGGATCGTTTCGCATGATTGAACAAGATGGATTGCACGCAGGTTCTCCGGCCGCTTGGGTGGAGAGGCTATTCGGCTATGACTGGGCACAACAGACAATCGGCTGCTCTGATGCCGCCGTGTTCCGGCTGTCAGCGCAGGGGCGCCCGGTTCTTTTTGTCAAGACCGACCTGTCCGGTGCCCTGAATGAACTGCAGGACGAGGCAGCGCGGCTATCGTGGCTGGCCACGACGGGCGTTCCTTGCGCAGCTGTGCTCGACGTTGTCACTGAAGCGGGAAGGGACTGGCTGCTATTGGGCGAAGTGCCGGGGCAGGATCTCCTGTCATCTCACCTTGCTCCTGCCGAGAAAGTATCCATCATGGCTGATGCAATGCGGCGGCTGCATACGCTTGATCCGGCTACCTGCCCATTCGACCACCAAGCGAAACATCGCATCGAGCGAGCACGTACTCGGATGGAAGCCGGTCTTGTCGATCAGGATGATCTGGACGAAGAGCATCAGGGGCTCGCGCCAGCCGAACTGTTCGCCAGGCTCAAGGCGCGCATGCCCGACGGCGAGGATCTCGTCGTGACCCATGGCGATGCCTGCTTGCCGAATATCATGGTGGAAAATGGCCGCTTTTCTGGATTCATCGACTGTGGCCGGCTGGGTGTGGCGGACCGCTATCAGGACATAGCGTTGGCTACCCGTGATATTGCTGAAGAGCTTGGCGGCGAATGGGCTGACCGCTTCCTCGTGCTTTACGGTATCGCCGCTCCCGATTCGCAGCGCATCGCCTTCTATCGCCTTCTTGACGAGTTCTTCTGAGCGGGACTCTGGGGTTCGAAATGACCGACCAAGCGACGCCCAACCTGCCATCACGAGATTTCGATTCCACCGCCGCCTTCTATGAAAGGTTGGGCTTCGGAATCGTTTTCCGGGACGCCGGCTGGATGATCCTCCAGCGCGGGGATCTCATGCTGGAGTTCTTCGCCCACCCCAACTTGTTTATTGCAGCTTATAATGGTTACAAATAAAGCAATAGCATCACAAATTTCACAAATAAAGCATTTTTTTCACTGCATTCTAGTTGTGGTTTGTCCAAACTCATCAATGTATCTTATCATGTCTGTATACCGTCGACCTCTAGCTAGAGCTTGGCGTAATCATGGTCATAGCTGTTTCCTGTGTGAAATTGTTATCCGCTCACAATTCCACACAACATACGAGCCGGAAGCATAAAGTGTAAAGCCTGGGGTGCCTAATGAGTGAGCTAACTCACATTAATTGCGTTGCGCTCACTGCCCGCTTTCCAGTCGGGAAACCTGTCGTGCCAGCTGCATTAATGAATCGGCCAACGCGCGGGGAGAGGCGGTTTGCGTATTGGGCGCTCTTCCGCTTCCTCGCTCACTGACTCGCTGCGCTCGGTCGTTCGGCTGCGGCGAGCGGTATCAGCTCACTCAAAGGCGGTAATACGGTTATCCACAGAATCAGGGGATAACGCAGGAAAGAACATGTGAGCAAAAGGCCAGCAAAAGGCCAGGAACCGTAAAAAGGCCGCGTTGCTGGCGTTTTTCCATAGGCTCCGCCCCCCTGACGAGCATCACAAAAATCGACGCTCAAGTCAGAGGTGGCGAAACCCGACAGGACTATAAAGATACCAGGCGTTTCCCCCTGGAAGCTCCCTCGTGCGCTCTCCTGTTCCGACCCTGCCGCTTACCGGATACCTGTCCGCCTTTCTCCCTTCGGGAAGCGTGGCGCTTTCTCATAGCTCACGCTGTAGGTATCTCAGTTCGGTGTAGGTCGTTCGCTCCAAGCTGGGCTGTGTGCACGAACCCCCCGTTCAGCCCGACCGCTGCGCCTTATCCGGTAACTATCGTCTTGAGTCCAACCCGGTAAGACACGACTTATCGCCACTGGCAGCAGCCACTGGTAACAGGATTAGCAGAGCGAGGTATGTAGGCGGTGCTACAGAGTTCTTGAAGTGGTGGCCTAACTACGGCTACACTAGAAGAACAGTATTTGGTATCTGCGCTCTGCTGAAGCCAGTTACCTTCGGAAAAAGAGTTGGTAGCTCTTGATCCGGCAAACAAACCACCGCTGGTAGCGGTGGTTTTTTTGTTTGCAAGCAGCAGATTACGCGCAGAAAAAAAGGATCTCAAGAAGATCCTTTGATCTTTTCTACGGGGTCTGACGCTCAGTGGAACGAAAACTCACGTTAAGGGATTTTGGTCATGAGATTATCAAAAAGGATCTTCACCTAGATCCTTTTAAATTAAAAATGAAGTTTTAAATCAATCTAAAGTATATATGAGTAAACTTGGTCTGACAGTTACCAATGCTTAATCAGTGAGGCACCTATCTCAGCGATCTGTCTATTTCGTTCATCCATAGTTGCCTGACTCCCCGTCGTGTAGATAACTACGATACGGGAGGGCTTACCATCTGGCCCCAGTGCTGCAATGATACCGCGAGACCCACGCTCACCGGCTCCAGATTTATCAGCAATAAACCAGCCAGCCGGAAGGGCCGAGCGCAGAAGTGGTCCTGCAACTTTATCCGCCTCCATCCAGTCTATTAATTGTTGCCGGGAAGCTAGAGTAAGTAGTTCGCCAGTTAATAGTTTGCGCAACGTTGTTGCCATTGCTACAGGCATCGTGGTGTCACGCTCGTCGTTTGGTATGGCTTCATTCAGCTCCGGTTCCCAACGATCAAGGCGAGTTACATGATCCCCCATGTTGTGCAAAAAAGCGGTTAGCTCCTTCGGTCCTCCGATCGTTGTCAGAAGTAAGTTGGCCGCAGTGTTATCACTCATGGTTATGGCAGCACTGCATAATTCTCTTACTGTCATGCCATCCGTAAGATGCTTTTCTGTGACTGGTGAGTACTCAACCAAGTCATTCTGAGAATAGTGTATGCGGCGACCGAGTTGCTCTTGCCCGGCGTCAATACGGGATAATACCGCGCCACATAGCAGAACTTTAAAAGTGCTCATCATTGGAAAACGTTCTTCGGGGCGAAAACTCTCAAGGATCTTACCGCTGTTGAGATCCAGTTCGATGTAACCCACTCGTGCACCCAACTGATCTTCAGCATCTTTTACTTTCACCAGCGTTTCTGGGTGAGCAAAAACAGGAAGGCAAAATGCCGCAAAAAAGGGAATAAGGGCGACACGGAAATGTTGAATACTCATACTCTTCCTTTTTCAATATTATTGAAGCATTTATCAGGGTTATTGTCTCATGAGCGGATACATATTTGAATGTATTTAGAAAAATAAACAAATAGGGGTTCCGCGCACATTTCCCCGAAAAGTGCCACCTGACGTC |
| PDK1-eGFP | TAGTTATTAATAGTAATCAATTACGGGGTCATTAGTTCATAGCCCATATATGGAGTTCCGCGTTACATAACTTACGGTAAATGGCCCGCCTGGCTGACCGCCCAACGACCCCCGCCCATTGACGTCAATAATGACGTATGTTCCCATAGTAACGCCAATAGGGACTTTCCATTGACGTCAATGGGTGGAGTATTTACGGTAAACTGCCCACTTGGCAGTACATCAAGTGTATCATATGCCAAGTACGCCCCCTATTGACGTCAATGACGGTAAATGGCCCGCCTGGCATTATGCCCAGTACATGACCTTATGGGACTTTCCTACTTGGCAGTACATCTACGTATTAGTCATCGCTATTACCATGGTGATGCGGTTTTGGCAGTACATCAATGGGCGTGGATAGCGGTTTGACTCACGGGGATTTCCAAGTCTCCACCCCATTGACGTCAATGGGAGTTTGTTTTGGCACCAAAATCAACGGGACTTTCCAAAATGTCGTAACAACTCCGCCCCATTGACGCAAATGGGCGGTAGGCGTGTACGGTGGGAGGTCTATATAAGCAGAGCTGGTTTAGTGAACCGTCAGATCCGCTAGCGCTACCGGTCGCCACCATGGCCAGGACCACCAGCCAGCTGTATGACGCCGTGCCCATCCAGTCCAGCGTGGTGTTATGTTCCTGCCCATCCCCATCAATGGTGAGGACCCAGACTGAGTCCAGCACGCCCCCTGGCATTCCTGGTGGCAGCAGGCAGGGCCCCGCCATGGACGGCACTGCAGCCGAGCCTCGGCCCGGCGCCGGCTCCCTGCAGCATGCCCAGCCTCCGCCGCAGCCTCGGAAGAAGCGGCCTGAGGACTTCAAGTTTGGGAAAATCCTTGGGGAAGGCTCTTTTTCCACGGTTGTCCTGGCTCGAGAACTGGCAACCTCCAGAGAATATGCGATTAAAATTCTGGAGAAGCGACATATCATAAAAGAGAACAAGGTCCCCTATGTAACCAGAGAGCGGGATGTCATGTCGCGCCTGGATCACCCCTTCTTTGTTAAGCTTTACTTCACATTTCAGGACGACGAGAAGCTGTATTTCGGCCTTAGTTATGCCAAAAATGGAGAACTACTTAAATATATTCGCAAAATCGGTTCATTCGATGAGACCTGTACCCGATTTTACACGGCTGAGATTGTGTCTGCTTTAGAGTACTTGCACGGCAAGGGCATCATTCACAGGGACCTTAAACCGGAAAACATTTTGTTAAATGAAGATATGCACATCCAGATCACAGATTTTGGAACAGCAAAAGTCTTATCCCCAGAGAGCAAACAAGCCAGGGCCAACTCATTCGTGGGAACAGCGCAGTACGTTTCTCCAGAGCTGCTCACGGAGAAGTCCGCCTGTAAGAGTTCAGACCTTTGGGCTCTTGGATGCATAATATACCAGCTTGTGGCAGGACTCCCACCATTCCGAGCTGGAAACGAGTATCTTATATTTCAGAAGATCATTAAGTTGGAATATGACTTTCCAGAAAAATTCTTCCCTAAGGCAAGAGACCTCGTGGAGAAACTTTTGGTTTTAGATGCCACAAAGCGGTTAGGCTGTGAGGAAATGGAAGGATACGGACCTCTTAAAGCACACCCGTTCTTCGAGTCCGTCACGTGGGAGAACCTGCACCAGCAGACGCCTCCGAAGCTCACCGCTTACCTGCCGGCTATGTCGGAAGACGACGAGGACTGCTATGGCAATTATGACAATCTCCTGAGCCAGTTTGGCTGCATGCAGGTGTCTTCGTCCTCCTCCTCACACTCCCTGTCAGCCTCCGACACGGGCCTGCCCCAGAGGTCAGGCAGCAACATAGAGCAGTACATTCACGATCTGGACTCGAACTCCTTTGAACTGGACTTACAGTTTTCCGAAGATGAGAAGAGGTTGTTGTTGGAGAAGCAGGCTGGCGGAAACCCTTGGCACCAGTTTGTAGAAAATAATTTAATACTAAAGATGGGCCCAGTGGATAAGCGGAAGGGTTTATTTGCAAGACGACGACAGCTGTTGCTCACAGAAGGACCACATTTATATTATGTGGATCCTGTCAACAAAGTTCTGAAAGGTGAAATTCCTTGGTCACAAGAACTTCGACCAGAGGCCAAGAATTTTAAAACTTTCTTTGTCCACACGCCTAACAGGACGTATTATCTGATGGACCCCAGCGGGAACGCACACAAGTGGTGCAGGAAGATCCAGGAGGTTTGGAGGCAGCGATACCAGAGCCACCCGGACGCCGCTGTGCAGGTGAGCAAGGGCGAGGAGCTGTTCACCGGGGTGGTGCCCATCCTGGTCGAGCTGGACGGCGACGTAAACGGCCACAAGTTCAGCGTGTCCGGCGAGGGCGAGGGCGATGCCACCTACGGCAAGCTGACCCTGAAGTTCATCTGCACCACCGGCAAGCTGCCCGTGCCCTGGCCCACCCTCGTGACCACCCTGACCTACGGCGTGCAGTGCTTCAGCCGCTACCCCGACCACATGAAGCAGCACGACTTCTTCAAGTCCGCCATGCCCGAAGGCTACGTCCAGGAGCGCACCATCTTCTTCAAGGACGACGGCAACTACAAGACCCGCGCCGAGGTGAAGTTCGAGGGCGACACCCTGGTGAACCGCATCGAGCTGAAGGGCATCGACTTCAAGGAGGACGGCAACATCCTGGGGCACAAGCTGGAGTACAACTACAACAGCCACAACGTCTATATCATGGCCGACAAGCAGAAGAACGGCATCAAGGTGAACTTCAAGATCCGCCACAACATCGAGGACGGCAGCGTGCAGCTCGCCGACCACTACCAGCAGAACACCCCCATCGGCGACGGCCCCGTGCTGCTGCCCGACAACCACTACCTGAGCACCCAGTCCGCCCTGAGCAAAGACCCCAACGAGAAGCGCGATCACATGGTCCTGCTGGAGTTCGTGACCGCCGCCGGGATCACTCTCGGCATGGACGAGCTGTACAAGTCCGGACTCAGATCTCGAGCTCAAGCTTCGAATTCTGCAGTCGACGGTACCGCGGGCCCGGGATCCACCGGATCTAGATAACTGATCATAATCAGCCATACCACATTTGTAGAGGTTTTACTTGCTTTAAAAAACCTCCCACACCTCCCCCTGAACCTGAAACATAAAATGAATGCAATTGTTGTTGTTAACTTGTTTATTGCAGCTTATAATGGTTACAAATAAAGCAATAGCATCACAAATTTCACAAATAAAGCATTTTTTTCACTGCATTCTAGTTGTGGTTTGTCCAAACTCATCAATGTATCTTAACGCGTAAATTGTAAGCGTTAATATTTTGTTAAAATTCGCGTTAAATTTTTGTTAAATCAGCTCATTTTTTAACCAATAGGCCGAAATCGGCAAAATCCCTTATAAATCAAAAGAATAGACCGAGATAGGGTTGAGTGTTGTTCCAGTTTGGAACAAGAGTCCACTATTAAAGAACGTGGACTCCAACGTCAAAGGGCGAAAAACCGTCTATCAGGGCGATGGCCCACTACGTGAACCATCACCCTAATCAAGTTTTTTGGGGTCGAGGTGCCGTAAAGCACTAAATCGGAACCCTAAAGGGAGCCCCCGATTTAGAGCTTGACGGGGAAAGCCGGCGAACGTGGCGAGAAAGGAAGGGAAGAAAGCGAAAGGAGCGGGCGCTAGGGCGCTGGCAAGTGTAGCGGTCACGCTGCGCGTAACCACCACACCCGCCGCGCTTAATGCGCCGCTACAGGGCGCGTCAGGTGGCACTTTTCGGGGAAATGTGCGCGGAACCCCTATTTGTTTATTTTTCTAAATACATTCAAATATGTATCCGCTCATGAGACAATAACCCTGATAAATGCTTCAATAATATTGAAAAAGGAAGAGTCCTGAGGCGGAAAGAACCAGCTGTGGAATGTGTGTCAGTTAGGGTGTGGAAAGTCCCCAGGCTCCCCAGCAGGCAGAAGTATGCAAAGCATGCATCTCAATTAGTCAGCAACCAGGTGTGGAAAGTCCCCAGGCTCCCCAGCAGGCAGAAGTATGCAAAGCATGCATCTCAATTAGTCAGCAACCATAGTCCCGCCCCTAACTCCGCCCATCCCGCCCCTAACTCCGCCCAGTTCCGCCCATTCTCCGCCCCATGGCTGACTAATTTTTTTTATTTATGCAGAGGCCGAGGCCGCCTCGGCCTCTGAGCTATTCCAGAAGTAGTGAGGAGGCTTTTTTGGAGGCCTAGGCTTTTGCAAAGATCGATCAAGAGACAGGATGAGGATCGTTTCGCATGATTGAACAAGATGGATTGCACGCAGGTTCTCCGGCCGCTTGGGTGGAGAGGCTATTCGGCTATGACTGGGCACAACAGACAATCGGCTGCTCTGATGCCGCCGTGTTCCGGCTGTCAGCGCAGGGGCGCCCGGTTCTTTTTGTCAAGACCGACCTGTCCGGTGCCCTGAATGAACTGCAAGACGAGGCAGCGCGGCTATCGTGGCTGGCCACGACGGGCGTTCCTTGCGCAGCTGTGCTCGACGTTGTCACTGAAGCGGGAAGGGACTGGCTGCTATTGGGCGAAGTGCCGGGGCAGGATCTCCTGTCATCTCACCTTGCTCCTGCCGAGAAAGTATCCATCATGGCTGATGCAATGCGGCGGCTGCATACGCTTGATCCGGCTACCTGCCCATTCGACCACCAAGCGAAACATCGCATCGAGCGAGCACGTACTCGGATGGAAGCCGGTCTTGTCGATCAGGATGATCTGGACGAAGAGCATCAGGGGCTCGCGCCAGCCGAACTGTTCGCCAGGCTCAAGGCGAGCATGCCCGACGGCGAGGATCTCGTCGTGACCCATGGCGATGCCTGCTTGCCGAATATCATGGTGGAAAATGGCCGCTTTTCTGGATTCATCGACTGTGGCCGGCTGGGTGTGGCGGACCGCTATCAGGACATAGCGTTGGCTACCCGTGATATTGCTGAAGAGCTTGGCGGCGAATGGGCTGACCGCTTCCTCGTGCTTTACGGTATCGCCGCTCCCGATTCGCAGCGCATCGCCTTCTATCGCCTTCTTGACGAGTTCTTCTGAGCGGGACTCTGGGGTTCGAAATGACCGACCAAGCGACGCCCAACCTGCCATCACGAGATTTCGATTCCACCGCCGCCTTCTATGAAAGGTTGGGCTTCGGAATCGTTTTCCGGGACGCCGGCTGGATGATCCTCCAGCGCGGGGATCTCATGCTGGAGTTCTTCGCCCACCCTAGGGGGAGGCTAACTGAAACACGGAAGGAGACAATACCGGAAGGAACCCGCGCTATGACGGCAATAAAAAGACAGAATAAAACGCACGGTGTTGGGTCGTTTGTTCATAAACGCGGGGTTCGGTCCCAGGGCTGGCACTCTGTCGATACCCCACCGAGACCCCATTGGGGCCAATACGCCCGCGTTTCTTCCTTTTCCCCACCCCACCCCCCAAGTTCGGGTGAAGGCCCAGGGCTCGCAGCCAACGTCGGGGCGGCAGGCCCTGCCATAGCCTCAGGTTACTCATATATACTTTAGATTGATTTAAAACTTCATTTTTAATTTAAAAGGATCTAGGTGAAGATCCTTTTTGATAATCTCATGACCAAAATCCCTTAACGTGAGTTTTCGTTCCACTGAGCGTCAGACCCCGTAGAAAAGATCAAAGGATCTTCTTGAGATCCTTTTTTTCTGCGCGTAATCTGCTGCTTGCAAACAAAAAAACCACCGCTACCAGCGGTGGTTTGTTTGCCGGATCAAGAGCTACCAACTCTTTTTCCGAAGGTAACTGGCTTCAGCAGAGCGCAGATACCAAATACTGTCCTTCTAGTGTAGCCGTAGTTAGGCCACCACTTCAAGAACTCTGTAGCACCGCCTACATACCTCGCTCTGCTAATCCTGTTACCAGTGGCTGCTGCCAGTGGCGATAAGTCGTGTCTTACCGGGTTGGACTCAAGACGATAGTTACCGGATAAGGCGCAGCGGTCGGGCTGAACGGGGGGTTCGTGCACACAGCCCAGCTTGGAGCGAACGACCTACACCGAACTGAGATACCTACAGCGTGAGCTATGAGAAAGCGCCACGCTTCCCGAAGGGAGAAAGGCGGACAGGTATCCGGTAAGCGGCAGGGTCGGAACAGGAGAGCGCACGAGGGAGCTTCCAGGGGGAAACGCCTGGTATCTTTATAGTCCTGTCGGGTTTCGCCACCTCTGACTTGAGCGTCGATTTTTGTGATGCTCGTCAGGGGGGCGGAGCCTATGGAAAAACGCCAGCAACGCGGCCTTTTTACGGTTCCTGGCCTTTTGCTGGCCTTTTGCTCACATGTTCTTTCCTGCGTTATCCCCTGATTCTGTGGATAACCGTATTACCGCCATGCAT |
| IRS1-eGFP | TAGTTATTAATAGTAATCAATTACGGGGTCATTAGTTCATAGCCCATATATGGAGTTCCGCGTTACATAACTTACGGTAAATGGCCCGCCTGGCTGACCGCCCAACGACCCCCGCCCATTGACGTCAATAATGACGTATGTTCCCATAGTAACGCCAATAGGGACTTTCCATTGACGTCAATGGGTGGAGTATTTACGGTAAACTGCCCACTTGGCAGTACATCAAGTGTATCATATGCCAAGTACGCCCCCTATTGACGTCAATGACGGTAAATGGCCCGCCTGGCATTATGCCCAGTACATGACCTTATGGGACTTTCCTACTTGGCAGTACATCTACGTATTAGTCATCGCTATTACCATGGTGATGCGGTTTTGGCAGTACATCAATGGGCGTGGATAGCGGTTTGACTCACGGGGATTTCCAAGTCTCCACCCCATTGACGTCAATGGGAGTTTGTTTTGGCACCAAAATCAACGGGACTTTCCAAAATGTCGTAACAACTCCGCCCCATTGACGCAAATGGGCGGTAGGCGTGTACGGTGGGAGGTCTATATAAGCAGAGCTGGTTTAGTGAACCGTCAGATCCGCTAGCGCTACCGGTCGCCACCATGGCGAGCCCTCCGGAGAGCGATGGCTTCTCGGACGTGCGCAAGGTGGGCTACCTGCGCAAACCCAAGAGCATGCACAAACGCTTCTTCGTACTGCGCGCGGCCAGCGAGGCTGGGGGCCCGGCGCGCCTCGAGTACTACGAGAACGAGAAGAAGTGGCGGCACAAGTCGAGCGCCCCCAAACGCTCGATCCCCCTTGAGAGCTGCTTCAACATCAACAAGCGGGCTGACTCCAAGAACAAGCACCTGGTGGCTCTCTACACCCGGGACGAGCACTTTGCCATCGCGGCGGACAGCGAGGCCGAGCAAGACAGCTGGTACCAGGCTCTCCTACAGCTGCACAACCGTGCTAAGGGCCACCACGACGGAGCTGCGGCCCTCGGGGCGGGAGGTGGTGGGGGCAGCTGCAGCGGCAGCTCCGGCCTTGGTGAGGCTGGGGAGGACTTGAGCTACGGTGACGTGCCCCCAGGACCCGCATTCAAAGAGGTCTGGCAAGTGATCCTGAAGCCCAAGGGCCTGGGTCAGACAAAGAACCTGATTGGTATCTACCGCCTTTGCCTGACCAGCAAGACCATCAGCTTCGTGAAGCTGAACTCGGAGGCAGCGGCCGTGGTGCTGCAGCTGATGAACATCAGGCGCTGTGGCCACTCGGAAAACTTCTTCTTCATCGAGGTGGGCCGTTCTGCCGTGACGGGGCCCGGGGAGTTCTGGATGCAGGTGGATGACTCTGTGGTGGCCCAGAACATGCACGAGACCATCCTGGAGGCCATGCGGGCCATGAGTGATGAGTTCCGCCCTCGCAGCAAGAGCCAGTCCTCGTCCAACTGCTCTAACCCCATCAGCGTCCCCCTGCGCCGGCACCATCTCAACAATCCCCCGCCCAGCCAGGTGGGGCTGACCCGCCGATCACGCACTGAGAGCATCACCGCCACCTCCCCGGCCAGCATGGTGGGCGGGAAGCCAGGCTCCTTCCGTGTCCGCGCCTCCAGTGACGGCGAAGGCACCATGTCCCGCCCAGCCTCGGTGGACGGCAGCCCTGTGAGTCCCAGCACCAACAGAACCCACGCCCACCGGCATCGGGGCAGCGCCCGGCTGCACCCCCCGCTCAACCACAGCCGCTCCATCCCCATGCCGGCTTCCCGCTGCTCGCCTTCGGCCACCAGCCCGGTCAGTCTGTCGTCCAGTAGCACCAGTGGCCATGGCTCCACCTCGGATTGTCTCTTCCCACGGCGATCTAGTGCTTCGGTGTCTGGTTCCCCCAGCGATGGCGGTTTCATCTCCTCGGATGAGTATGGCTCCAGTCCCTGCGATTTCCGGAGTTCCTTCCGCAGTGTCACTCCGGATTCCCTGGGCCACACCCCACCAGCCCGCGGTGAGGAGGAGCTAAGCAACTATATCTGCATGGGTGGCAAGGGGCCCTCCACCCTGACCGCCCCCAACGGTCACTACATTTTGTCTCGGGGTGGCAATGGCCACCGCTGCACCCCAGGAACAGGCTTGGGCACGAGTCCAGCCTTGGCTGGGGATGAAGCAGCCAGTGCTGCAGATCTGGATAATCGGTTCCGAAAGAGAACTCACTCGGCAGGCACATCCCCTACCATTACCCACCAGAAGACCCCGTCCCAGTCCTCAGTGGCTTCCATTGAGGAGTACACAGAGATGATGCCTGCCTACCCACCAGGAGGTGGCAGTGGAGGCCGACTGCCGGGACACAGGCACTCCGCCTTCGTGCCCACCCGCTCCTACCCAGAGGAGGGTCTGGAAATGCACCCCTTGGAGCGTCGGGGGGGGCACCACCGCCCAGACAGCTCCACCCTCCACACGGATGATGGCTACATGCCCATGTCCCCAGGGGTGGCCCCAGTGCCCAGTGGCCGAAAGGGCAGTGGAGACTATATGCCCATGAGCCCCAAGAGCGTATCTGCCCCACAGCAGATCATCAATCCCATCAGACGCCATCCCCAGAGAGTGGACCCCAATGGCTACATGATGATGTCCCCCAGCGGTGGCTGCTCTCCTGACATTGGAGGTGGCCCCAGCAGCAGCAGCAGCAGCAGCAACGCCGTCCCTTCCGGGACCAGCTATGGAAAGCTGTGGACAAACGGGGTAGGGGGCCACCACTCTCATGTCTTGCCTCACCCCAAACCCCCAGTGGAGAGCAGCGGTGGTAAGCTCTTACCTTGCACAGGTGACTACATGAACATGTCACCAGTGGGGGACTCCAACACCAGCAGCCCCTCCGACTGCTACTACGGCCCTGAGGACCCCCAGCACAAGCCAGTCCTCTCCTACTACTCATTGCCAAGATCCTTTAAGCACACCCAGCGCCCCGGGGAGCCGGAGGAGGGTGCCCGGCATCAGCACCTCCGCCTTTCCACTAGCTCTGGTCGCCTTCTCTATGCTGCAACAGCAGATGATTCTTCCTCTTCCACCAGCAGCGACAGCCTGGGTGGGGGATACTGCGGGGCTAGGCTGGAGCCCAGCCTTCCACATCCCCACCATCAGGTTCTGCAGCCCCATCTGCCTCGAAAGGTGGACACAGCTGCTCAGACCAATAGCCGCCTGGCCCGGCCCACGAGGCTGTCCCTGGGGGATCCCAAGGCCAGCACCTTACCTCGGGCCCGAGAGCAGCAGCAGCAGCAGCAGCCCTTGCTGCACCCTCCAGAGCCCAAGAGCCCGGGGGAATATGTCAATATTGAATTTGGGAGTGATCAGTCTGGCTACTTGTCTGGCCCGGTGGCTTTCCACAGCTCACCTTCTGTCAGGTGTCCATCCCAGCTCCAGCCAGCTCCCAGAGAGGAAGAGACTGGCACTGAGGAGTACATGAAGATGGACCTGGGGCCGGGCCGGAGGGCAGCCTGGCAGGAGAGCACTGGGGTCGAGATGGGCAGACTGGGCCCTGCACCTCCCGGGGCTGCTAGCATTTGCAGGCCTACCCGGGCAGTGCCCAGCAGCCGGGGTGACTACATGACCATGCAGATGAGTTGTCCCCGTCAGAGCTACGTGGACACCTCGCCAGCTGCCCCTGTAAGCTATGCTGACATGCGAACAGGCATTGCTGCAGAGGAGGTGAGCCTGCCCAGGGCTACTATGGCTGCTGCCTCCTCATCCTCAGCAGCCTCTGCTTCCCCGACTGGGCCTCAAGGGGCAGCAGAGCTGGCTGCCCACTCGTCCCTGCTGGGGGGCCCACAAGGACCTGGGGGCATGAGCGCCTTCACCCGGGTGAACCTCAGTCCTAACCGCAACCAGAGTGCCAAAGTGATCCGTGCAGACCCACAAGGGTGCCGGCGGAGGCATAGCTCCGAGACTTTCTCCTCAACACCCAGTGCCACCCGGGTGGGCAACACAGTGCCCTTTGGAGCGGGGGCAGCAGTAGGGGGCGGTGGCGGTAGCAGCAGCAGCAGCGAGGATGTGAAACGCCACAGCTCTGCTTCCTTTGAGAATGTGTGGCTGAGGCCTGGGGAGCTTGGGGGAGCCCCCAAGGAGCCAGCCAAACTGTGTGGGGCTGCTGGGGGTTTGGAGAATGGTCTTAACTACATAGACCTGGATTTGGTCAAGGACTTCAAACAGTGCCCTCAGGAGTGCACCCCTGAACCGCAGCCTCCCCCACCCCCACCCCCTCATCAACCCCTGGGCAGCGGTGAGAGCAGCTCCACCCGCCGCTCAAGTGAGGATTTAAGCGCCTATGCCAGCATCAGTTTCCAGAAGCAGCCAGAGGACCGTCAGGTGAGCAAGGGCGAGGAGCTGTTCACCGGGGTGGTGCCCATCCTGGTCGAGCTGGACGGCGACGTAAACGGCCACAAGTTCAGCGTGTCCGGCGAGGGCGAGGGCGATGCCACCTACGGCAAGCTGACCCTGAAGTTCATCTGCACCACCGGCAAGCTGCCCGTGCCCTGGCCCACCCTCGTGACCACCCTGACCTACGGCGTGCAGTGCTTCAGCCGCTACCCCGACCACATGAAGCAGCACGACTTCTTCAAGTCCGCCATGCCCGAAGGCTACGTCCAGGAGCGCACCATCTTCTTCAAGGACGACGGCAACTACAAGACCCGCGCCGAGGTGAAGTTCGAGGGCGACACCCTGGTGAACCGCATCGAGCTGAAGGGCATCGACTTCAAGGAGGACGGCAACATCCTGGGGCACAAGCTGGAGTACAACTACAACAGCCACAACGTCTATATCATGGCCGACAAGCAGAAGAACGGCATCAAGGTGAACTTCAAGATCCGCCACAACATCGAGGACGGCAGCGTGCAGCTCGCCGACCACTACCAGCAGAACACCCCCATCGGCGACGGCCCCGTGCTGCTGCCCGACAACCACTACCTGAGCACCCAGTCCGCCCTGAGCAAAGACCCCAACGAGAAGCGCGATCACATGGTCCTGCTGGAGTTCGTGACCGCCGCCGGGATCACTCTCGGCATGGACGAGCTGTACAAGTCCGGACTCAGATCTCGAGCTCAAGCTTCGAATTCTGCAGTCGACGGTACCGCGGGCCCGGGATCCACCGGATCTAGATAACTGATCATAATCAGCCATACCACATTTGTAGAGGTTTTACTTGCTTTAAAAAACCTCCCACACCTCCCCCTGAACCTGAAACATAAAATGAATGCAATTGTTGTTGTTAACTTGTTTATTGCAGCTTATAATGGTTACAAATAAAGCAATAGCATCACAAATTTCACAAATAAAGCATTTTTTTCACTGCATTCTAGTTGTGGTTTGTCCAAACTCATCAATGTATCTTAACGCGTAAATTGTAAGCGTTAATATTTTGTTAAAATTCGCGTTAAATTTTTGTTAAATCAGCTCATTTTTTAACCAATAGGCCGAAATCGGCAAAATCCCTTATAAATCAAAAGAATAGACCGAGATAGGGTTGAGTGTTGTTCCAGTTTGGAACAAGAGTCCACTATTAAAGAACGTGGACTCCAACGTCAAAGGGCGAAAAACCGTCTATCAGGGCGATGGCCCACTACGTGAACCATCACCCTAATCAAGTTTTTTGGGGTCGAGGTGCCGTAAAGCACTAAATCGGAACCCTAAAGGGAGCCCCCGATTTAGAGCTTGACGGGGAAAGCCGGCGAACGTGGCGAGAAAGGAAGGGAAGAAAGCGAAAGGAGCGGGCGCTAGGGCGCTGGCAAGTGTAGCGGTCACGCTGCGCGTAACCACCACACCCGCCGCGCTTAATGCGCCGCTACAGGGCGCGTCAGGTGGCACTTTTCGGGGAAATGTGCGCGGAACCCCTATTTGTTTATTTTTCTAAATACATTCAAATATGTATCCGCTCATGAGACAATAACCCTGATAAATGCTTCAATAATATTGAAAAAGGAAGAGTCCTGAGGCGGAAAGAACCAGCTGTGGAATGTGTGTCAGTTAGGGTGTGGAAAGTCCCCAGGCTCCCCAGCAGGCAGAAGTATGCAAAGCATGCATCTCAATTAGTCAGCAACCAGGTGTGGAAAGTCCCCAGGCTCCCCAGCAGGCAGAAGTATGCAAAGCATGCATCTCAATTAGTCAGCAACCATAGTCCCGCCCCTAACTCCGCCCATCCCGCCCCTAACTCCGCCCAGTTCCGCCCATTCTCCGCCCCATGGCTGACTAATTTTTTTTATTTATGCAGAGGCCGAGGCCGCCTCGGCCTCTGAGCTATTCCAGAAGTAGTGAGGAGGCTTTTTTGGAGGCCTAGGCTTTTGCAAAGATCGATCAAGAGACAGGATGAGGATCGTTTCGCATGATTGAACAAGATGGATTGCACGCAGGTTCTCCGGCCGCTTGGGTGGAGAGGCTATTCGGCTATGACTGGGCACAACAGACAATCGGCTGCTCTGATGCCGCCGTGTTCCGGCTGTCAGCGCAGGGGCGCCCGGTTCTTTTTGTCAAGACCGACCTGTCCGGTGCCCTGAATGAACTGCAAGACGAGGCAGCGCGGCTATCGTGGCTGGCCACGACGGGCGTTCCTTGCGCAGCTGTGCTCGACGTTGTCACTGAAGCGGGAAGGGACTGGCTGCTATTGGGCGAAGTGCCGGGGCAGGATCTCCTGTCATCTCACCTTGCTCCTGCCGAGAAAGTATCCATCATGGCTGATGCAATGCGGCGGCTGCATACGCTTGATCCGGCTACCTGCCCATTCGACCACCAAGCGAAACATCGCATCGAGCGAGCACGTACTCGGATGGAAGCCGGTCTTGTCGATCAGGATGATCTGGACGAAGAGCATCAGGGGCTCGCGCCAGCCGAACTGTTCGCCAGGCTCAAGGCGAGCATGCCCGACGGCGAGGATCTCGTCGTGACCCATGGCGATGCCTGCTTGCCGAATATCATGGTGGAAAATGGCCGCTTTTCTGGATTCATCGACTGTGGCCGGCTGGGTGTGGCGGACCGCTATCAGGACATAGCGTTGGCTACCCGTGATATTGCTGAAGAGCTTGGCGGCGAATGGGCTGACCGCTTCCTCGTGCTTTACGGTATCGCCGCTCCCGATTCGCAGCGCATCGCCTTCTATCGCCTTCTTGACGAGTTCTTCTGAGCGGGACTCTGGGGTTCGAAATGACCGACCAAGCGACGCCCAACCTGCCATCACGAGATTTCGATTCCACCGCCGCCTTCTATGAAAGGTTGGGCTTCGGAATCGTTTTCCGGGACGCCGGCTGGATGATCCTCCAGCGCGGGGATCTCATGCTGGAGTTCTTCGCCCACCCTAGGGGGAGGCTAACTGAAACACGGAAGGAGACAATACCGGAAGGAACCCGCGCTATGACGGCAATAAAAAGACAGAATAAAACGCACGGTGTTGGGTCGTTTGTTCATAAACGCGGGGTTCGGTCCCAGGGCTGGCACTCTGTCGATACCCCACCGAGACCCCATTGGGGCCAATACGCCCGCGTTTCTTCCTTTTCCCCACCCCACCCCCCAAGTTCGGGTGAAGGCCCAGGGCTCGCAGCCAACGTCGGGGCGGCAGGCCCTGCCATAGCCTCAGGTTACTCATATATACTTTAGATTGATTTAAAACTTCATTTTTAATTTAAAAGGATCTAGGTGAAGATCCTTTTTGATAATCTCATGACCAAAATCCCTTAACGTGAGTTTTCGTTCCACTGAGCGTCAGACCCCGTAGAAAAGATCAAAGGATCTTCTTGAGATCCTTTTTTTCTGCGCGTAATCTGCTGCTTGCAAACAAAAAAACCACCGCTACCAGCGGTGGTTTGTTTGCCGGATCAAGAGCTACCAACTCTTTTTCCGAAGGTAACTGGCTTCAGCAGAGCGCAGATACCAAATACTGTCCTTCTAGTGTAGCCGTAGTTAGGCCACCACTTCAAGAACTCTGTAGCACCGCCTACATACCTCGCTCTGCTAATCCTGTTACCAGTGGCTGCTGCCAGTGGCGATAAGTCGTGTCTTACCGGGTTGGACTCAAGACGATAGTTACCGGATAAGGCGCAGCGGTCGGGCTGAACGGGGGGTTCGTGCACACAGCCCAGCTTGGAGCGAACGACCTACACCGAACTGAGATACCTACAGCGTGAGCTATGAGAAAGCGCCACGCTTCCCGAAGGGAGAAAGGCGGACAGGTATCCGGTAAGCGGCAGGGTCGGAACAGGAGAGCGCACGAGGGAGCTTCCAGGGGGAAACGCCTGGTATCTTTATAGTCCTGTCGGGTTTCGCCACCTCTGACTTGAGCGTCGATTTTTGTGATGCTCGTCAGGGGGGCGGAGCCTATGGAAAAACGCCAGCAACGCGGCCTTTTTACGGTTCCTGGCCTTTTGCTGGCCTTTTGCTCACATGTTCTTTCCTGCGTTATCCCCTGATTCTGTGGATAACCGTATTACCGCCATGCAT |
| IRS2-eGFP | TAGTTATTAATAGTAATCAATTACGGGGTCATTAGTTCATAGCCCATATATGGAGTTCCGCGTTACATAACTTACGGTAAATGGCCCGCCTGGCTGACCGCCCAACGACCCCCGCCCATTGACGTCAATAATGACGTATGTTCCCATAGTAACGCCAATAGGGACTTTCCATTGACGTCAATGGGTGGAGTATTTACGGTAAACTGCCCACTTGGCAGTACATCAAGTGTATCATATGCCAAGTACGCCCCCTATTGACGTCAATGACGGTAAATGGCCCGCCTGGCATTATGCCCAGTACATGACCTTATGGGACTTTCCTACTTGGCAGTACATCTACGTATTAGTCATCGCTATTACCATGGTGATGCGGTTTTGGCAGTACATCAATGGGCGTGGATAGCGGTTTGACTCACGGGGATTTCCAAGTCTCCACCCCATTGACGTCAATGGGAGTTTGTTTTGGCACCAAAATCAACGGGACTTTCCAAAATGTCGTAACAACTCCGCCCCATTGACGCAAATGGGCGGTAGGCGTGTACGGTGGGAGGTCTATATAAGCAGAGCTGGTTTAGTGAACCGTCAGATCCGCTAGCGCTACCGGTCGCCACCATGGCTTCCCCCCCAAGGCACGGCCCCCCTGGACCTGCTTCTGGCGATGGACCTAATCTGAACAATAACAATAACAATAACAATCACTCCGTCCGGAAGTGCGGATACCTGAGAAAGCAGAAACATGGCCACAAACGATTCTTTGTGCTGCGAGGACCAGGAGCAGGAGGGGACGAGGCTACCGCAGGAGGAGGAAGCGCTCCTCAGCCACCTAGACTGGAATACTATGAAAGTGAGAAGAAATGGAGGTCAAAGGCAGGAGCTCCAAAACGCGTGATCGCACTGGACTGCTGTCTGAACATTAATAAGAGGGCAGATGCCAAGCACAAATACCTGATCGCCCTGTATACTAAAGATGAGTACTTTGCTGTCGCCGCTGAAAACGAGCAGGAACAGGAGGGCTGGTATCGAGCACTGACCGACCTGGTGTCCGAAGGACGAGCAGCAGCTGGAGATGCTCCACCAGCAGCAGCTCCAGCAGCATCATGCAGCGCATCCCTGCCAGGAGCTCTGGGAGGATCTGCAGGAGCTGCAGGAGCAGAGGACAGTTATGGACTGGTGGCTCCAGCAACAGCCGCTTACCGCGAAGTGTGGCAGGTCAACCTGAAGCCCAAAGGGCTGGGACAGTCTAAGAATCTGACCGGAGTGTACAGGCTGTGCCTGAGCGCACGCACAATCGGATTCGTGAAACTGAACTGTGAGCAGCCCAGCGTCACCCTGCAGCTGATGAATATCCGGAGATGTGGCCACTCTGATAGTTTCTTTTTCATTGAAGTGGGAAGATCCGCAGTCACAGGACCTGGGGAACTGTGGATGCAGGCTGACGATTCTGTGGTCGCACAGAACATCCATGAAACTATTCTGGAGGCCATGAAGGCTCTGAAAGAACTGTTTGAGTTCCGCCCCCGATCAAAGAGCCAGAGCTCCGGATCTAGTGCAACCCACCCTATTAGCGTGCCAGGAGCAAGGCGACACCATCACCTGGTCAATCTGCCTCCATCCCAGACAGGCCTGGTGCGACGGTCACGAACTGACAGCCTGGCAGCAACCCCACCTGCTGCAAAATGCTCAAGCTGTCGGGTGAGAACAGCCTCCGAGGGCGATGGGGGAGCAGCTGCAGGAGCAGCTGCAGCAGGAGCACGGCCTGTGTCTGTCGCTGGGAGTCCACTGTCACCAGGACCCGTGAGAGCCCCTCTGTCCAGGTCTCATACTCTGAGCGGAGGATGCGGAGGAAGAGGATCCAAGGTCGCACTGCTGCCAGCAGGAGGAGCACTGCAGCATAGTAGGTCAATGAGCATGCCAGTGGCACACAGCCCACCAGCTGCAACCAGCCCAGGATCCCTGTCCTCTAGTTCAGGACACGGCTCCGGATCTTATCCTCCACCACCTGGACCACATCCACCACTGCCTCACCCACTGCATCACGGACCAGGACAGAGGCCTAGCTCCGGAAGTGCATCAGCTAGCGGATCCCCATCTGATCCCGGCTTCATGTCCCTGGACGAGTACGGCTCTAGTCCTGGGGACCTGCGAGCCTTCTGCTCTCATCGGAGTAACACCCCAGAAAGCATCGCTGAGACACCTCCAGCAAGAGACGGAGGAGGAGGAGGAGAATTTTACGGCTATATGACAATGGATCGGCCACTGAGTCACTGTGGGAGATCATATAGAAGGGTGAGCGGAGACGCAGCTCAGGACCTGGATAGGGGCCTGAGGAAGCGCACTTACTCTCTGACCACACCAGCACGACAGCGACCAGTGCCTCAGCCATCAAGCGCTAGCCTGGATGAGTATACCCTGATGCGAGCAACATTCAGTGGCTCAGCCGGGAGGCTGTGCCCTTCCTGTCCAGCTTCCTCTCCCAAAGTGGCATACCATCCCTATCCTGAAGACTATGGAGATATCGAGATTGGCAGCCACCGGAGTTCAAGCTCCAATCTGGGAGCCGACGATGGCTACATGCCAATGACCCCTGGAGCAGCACTGGCTGGAAGCGGATCCGGCTCTTGTCGCAGTGACGATTACATGCCAATGTCACCCGCCAGTGTGTCAGCTCCCAAGCAGATCCTGCAGCCTCGAGCTGCAGCAGCTGCAGCAGCTGCAGTGCCATCCGCTGGACCAGCAGGACCAGCACCCACATCAGCCGCTGGCCGCACTTTTCCTGCCAGTGGAGGCGGGTATAAAGCATCTAGTCCTGCCGAATCAAGCCCAGAGGACAGCGGCTACATGCGGATGTGGTGCGGGTCAAAGCTGAGCATGGAGCATGCCGATGGCAAACTGCTGCCTAACGGGGACTACCTGAATGTGAGCCCATCCGATGCAGTCACTACCGGAACCCCACCTGACTTCTTCAGCGCAGCACTGCACCCTGGAGGAGAACCACTGCGAGGAGTGCCCGGATGCTGTTATTCCTCTCTGCCACGGAGCTACAAGGCCCCCTATACATGTGGGGGAGACTCCGATCAGTACGTGCTGATGAGTTCACCCGTCGGAAGAATTCTGGAGGAAGAGAGGCTGGAGCCTCAGGCAACTCCTGGACCATCCCAGGCTGCATCTGCTTTTGGGGCAGGACCTACCCAGCCACCACATCCAGTGGTCCCATCCCCTGTCCGCCCATCTGGAGGACGACCTGAGGGCTTCCTGGGACAGAGAGGAAGGGCCGTGAGGCCTACACGACTGAGCCTGGAGGGCCTGCCTTCCCTGCCATCTATGCACGAATATCCACTGCCTCCAGAGCCCAAGTCCCCTGGCGAATACATCAACATTGACTTTGGCGAGCCAGGAGCAAGACTGTCCCCACCTGCACCACCACTGCTGGCTTCTGCAGCTAGCTCCTCTAGTCTGCTGAGCGCTTCAAGCCCAGCATCCTCTCTGGGATCCGGAACACCTGGCACTAGTTCAGACTCTCGCCAGCGAAGTCCACTGTCAGATTATATGAATCTGGACTTCAGCTCCCCAAAGTCCCCAAAACCAGGAGCACCAAGCGGACACCCTGTGGGATCCCTGGATGGACTGCTGTCTCCAGAGGCCTCTAGTCCCTATCCTCCACTGCCCCCTAGACCATCTGCTAGTCCCTCAAGCTCCCTGCAGCCACCTCCTCCACCACCTGCACCAGGAGAACTGTACAGGCTGCCACCAGCAAGCGCAGTGGCTACCGCACAGGGACCTGGAGCAGCATCTAGTCTGTCAAGCGACACTGGAGATAACGGCGACTACACCGAGATGGCCTTTGGCGTGGCTGCAACTCCTCCACAGCCCATCGCCGCTCCCCCTAAGCCTGAAGCAGCAAGAGTGGCAAGCCCTACCTCCGGAGTCAAAAGGCTGAGTCTGATGGAGCAGGTGTCAGGGGTCGAAGCCTTCCTGCAGGCTTCCCAGCCACCAGACCCTCATCGAGGCGCAAAAGTGATCCGAGCAGATCCACAGGGAGGACGACGACGACACTCCTCTGAGACCTTCAGCTCAACAACTACCGTGACTCCAGTCTCACCCAGCTTCGCTCATAACCCCAAGAGACACAATTCCGCATCTGTGGAGAATGTCTCTCTGAGGAAAAGCTCCGAAGGAGGAGTGGGAGTCGGACCAGGAGGAGGAGACGAGCCTCCAACCAGCCCTCGACAGCTGCAGCCAGCACCCCCTCTGGCCCCCCAGGGACGGCCTTGGACACCAGGACAGCCAGGAGGACTGGTGGGATGCCCAGGATCTGGAGGAAGTCCTATGAGAAGGGAGACCAGCGCCGGATTCCAGAACGGCCTGAATTACATCGCTATTGACGTGCGGGAAGAGCCAGGACTGCCACCACAGCCACAGCCACCACCACCTCCACTGCCTCAGCCAGGGGATAAGTCTAGTTGGGGACGGACAAGAAGCCTGGGAGGACTGATCTCCGCAGTGGGAGTCGGATCTACTGGAGGAGGATGTGGAGGACCAGGACCTGGAGCTCTGCCCCCTGCAAATACCTACGCATCCATTGACTTTCTGTCCCATCATCTGAAGGAAGCAACTATCGTGAAGGAAGTGAGCAAGGGCGAGGAGCTGTTCACCGGGGTGGTGCCCATCCTGGTCGAGCTGGACGGCGACGTAAACGGCCACAAGTTCAGCGTGTCCGGCGAGGGCGAGGGCGATGCCACCTACGGCAAGCTGACCCTGAAGTTCATCTGCACCACCGGCAAGCTGCCCGTGCCCTGGCCCACCCTCGTGACCACCCTGACCTACGGCGTGCAGTGCTTCAGCCGCTACCCCGACCACATGAAGCAGCACGACTTCTTCAAGTCCGCCATGCCCGAAGGCTACGTCCAGGAGCGCACCATCTTCTTCAAGGACGACGGCAACTACAAGACCCGCGCCGAGGTGAAGTTCGAGGGCGACACCCTGGTGAACCGCATCGAGCTGAAGGGCATCGACTTCAAGGAGGACGGCAACATCCTGGGGCACAAGCTGGAGTACAACTACAACAGCCACAACGTCTATATCATGGCCGACAAGCAGAAGAACGGCATCAAGGTGAACTTCAAGATCCGCCACAACATCGAGGACGGCAGCGTGCAGCTCGCCGACCACTACCAGCAGAACACCCCCATCGGCGACGGCCCCGTGCTGCTGCCCGACAACCACTACCTGAGCACCCAGTCCGCCCTGAGCAAAGACCCCAACGAGAAGCGCGATCACATGGTCCTGCTGGAGTTCGTGACCGCCGCCGGGATCACTCTCGGCATGGACGAGCTGTACAAGTCCGGACTCAGATCTCGAGCTCAAGCTTCGAATTCTGCAGTCGACGGTACCGCGGGCCCGGGATCCACCGGATCTAGATAACTGATCATAATCAGCCATACCACATTTGTAGAGGTTTTACTTGCTTTAAAAAACCTCCCACACCTCCCCCTGAACCTGAAACATAAAATGAATGCAATTGTTGTTGTTAACTTGTTTATTGCAGCTTATAATGGTTACAAATAAAGCAATAGCATCACAAATTTCACAAATAAAGCATTTTTTTCACTGCATTCTAGTTGTGGTTTGTCCAAACTCATCAATGTATCTTAACGCGTAAATTGTAAGCGTTAATATTTTGTTAAAATTCGCGTTAAATTTTTGTTAAATCAGCTCATTTTTTAACCAATAGGCCGAAATCGGCAAAATCCCTTATAAATCAAAAGAATAGACCGAGATAGGGTTGAGTGTTGTTCCAGTTTGGAACAAGAGTCCACTATTAAAGAACGTGGACTCCAACGTCAAAGGGCGAAAAACCGTCTATCAGGGCGATGGCCCACTACGTGAACCATCACCCTAATCAAGTTTTTTGGGGTCGAGGTGCCGTAAAGCACTAAATCGGAACCCTAAAGGGAGCCCCCGATTTAGAGCTTGACGGGGAAAGCCGGCGAACGTGGCGAGAAAGGAAGGGAAGAAAGCGAAAGGAGCGGGCGCTAGGGCGCTGGCAAGTGTAGCGGTCACGCTGCGCGTAACCACCACACCCGCCGCGCTTAATGCGCCGCTACAGGGCGCGTCAGGTGGCACTTTTCGGGGAAATGTGCGCGGAACCCCTATTTGTTTATTTTTCTAAATACATTCAAATATGTATCCGCTCATGAGACAATAACCCTGATAAATGCTTCAATAATATTGAAAAAGGAAGAGTCCTGAGGCGGAAAGAACCAGCTGTGGAATGTGTGTCAGTTAGGGTGTGGAAAGTCCCCAGGCTCCCCAGCAGGCAGAAGTATGCAAAGCATGCATCTCAATTAGTCAGCAACCAGGTGTGGAAAGTCCCCAGGCTCCCCAGCAGGCAGAAGTATGCAAAGCATGCATCTCAATTAGTCAGCAACCATAGTCCCGCCCCTAACTCCGCCCATCCCGCCCCTAACTCCGCCCAGTTCCGCCCATTCTCCGCCCCATGGCTGACTAATTTTTTTTATTTATGCAGAGGCCGAGGCCGCCTCGGCCTCTGAGCTATTCCAGAAGTAGTGAGGAGGCTTTTTTGGAGGCCTAGGCTTTTGCAAAGATCGATCAAGAGACAGGATGAGGATCGTTTCGCATGATTGAACAAGATGGATTGCACGCAGGTTCTCCGGCCGCTTGGGTGGAGAGGCTATTCGGCTATGACTGGGCACAACAGACAATCGGCTGCTCTGATGCCGCCGTGTTCCGGCTGTCAGCGCAGGGGCGCCCGGTTCTTTTTGTCAAGACCGACCTGTCCGGTGCCCTGAATGAACTGCAAGACGAGGCAGCGCGGCTATCGTGGCTGGCCACGACGGGCGTTCCTTGCGCAGCTGTGCTCGACGTTGTCACTGAAGCGGGAAGGGACTGGCTGCTATTGGGCGAAGTGCCGGGGCAGGATCTCCTGTCATCTCACCTTGCTCCTGCCGAGAAAGTATCCATCATGGCTGATGCAATGCGGCGGCTGCATACGCTTGATCCGGCTACCTGCCCATTCGACCACCAAGCGAAACATCGCATCGAGCGAGCACGTACTCGGATGGAAGCCGGTCTTGTCGATCAGGATGATCTGGACGAAGAGCATCAGGGGCTCGCGCCAGCCGAACTGTTCGCCAGGCTCAAGGCGAGCATGCCCGACGGCGAGGATCTCGTCGTGACCCATGGCGATGCCTGCTTGCCGAATATCATGGTGGAAAATGGCCGCTTTTCTGGATTCATCGACTGTGGCCGGCTGGGTGTGGCGGACCGCTATCAGGACATAGCGTTGGCTACCCGTGATATTGCTGAAGAGCTTGGCGGCGAATGGGCTGACCGCTTCCTCGTGCTTTACGGTATCGCCGCTCCCGATTCGCAGCGCATCGCCTTCTATCGCCTTCTTGACGAGTTCTTCTGAGCGGGACTCTGGGGTTCGAAATGACCGACCAAGCGACGCCCAACCTGCCATCACGAGATTTCGATTCCACCGCCGCCTTCTATGAAAGGTTGGGCTTCGGAATCGTTTTCCGGGACGCCGGCTGGATGATCCTCCAGCGCGGGGATCTCATGCTGGAGTTCTTCGCCCACCCTAGGGGGAGGCTAACTGAAACACGGAAGGAGACAATACCGGAAGGAACCCGCGCTATGACGGCAATAAAAAGACAGAATAAAACGCACGGTGTTGGGTCGTTTGTTCATAAACGCGGGGTTCGGTCCCAGGGCTGGCACTCTGTCGATACCCCACCGAGACCCCATTGGGGCCAATACGCCCGCGTTTCTTCCTTTTCCCCACCCCACCCCCCAAGTTCGGGTGAAGGCCCAGGGCTCGCAGCCAACGTCGGGGCGGCAGGCCCTGCCATAGCCTCAGGTTACTCATATATACTTTAGATTGATTTAAAACTTCATTTTTAATTTAAAAGGATCTAGGTGAAGATCCTTTTTGATAATCTCATGACCAAAATCCCTTAACGTGAGTTTTCGTTCCACTGAGCGTCAGACCCCGTAGAAAAGATCAAAGGATCTTCTTGAGATCCTTTTTTTCTGCGCGTAATCTGCTGCTTGCAAACAAAAAAACCACCGCTACCAGCGGTGGTTTGTTTGCCGGATCAAGAGCTACCAACTCTTTTTCCGAAGGTAACTGGCTTCAGCAGAGCGCAGATACCAAATACTGTCCTTCTAGTGTAGCCGTAGTTAGGCCACCACTTCAAGAACTCTGTAGCACCGCCTACATACCTCGCTCTGCTAATCCTGTTACCAGTGGCTGCTGCCAGTGGCGATAAGTCGTGTCTTACCGGGTTGGACTCAAGACGATAGTTACCGGATAAGGCGCAGCGGTCGGGCTGAACGGGGGGTTCGTGCACACAGCCCAGCTTGGAGCGAACGACCTACACCGAACTGAGATACCTACAGCGTGAGCTATGAGAAAGCGCCACGCTTCCCGAAGGGAGAAAGGCGGACAGGTATCCGGTAAGCGGCAGGGTCGGAACAGGAGAGCGCACGAGGGAGCTTCCAGGGGGAAACGCCTGGTATCTTTATAGTCCTGTCGGGTTTCGCCACCTCTGACTTGAGCGTCGATTTTTGTGATGCTCGTCAGGGGGGCGGAGCCTATGGAAAAACGCCAGCAACGCGGCCTTTTTACGGTTCCTGGCCTTTTGCTGGCCTTTTGCTCACATGTTCTTTCCTGCGTTATCCCCTGATTCTGTGGATAACCGTATTACCGCCATGCAT |
| IRS2-FLAG | TAGTTATTAATAGTAATCAATTACGGGGTCATTAGTTCATAGCCCATATATGGAGTTCCGCGTTACATAACTTACGGTAAATGGCCCGCCTGGCTGACCGCCCAACGACCCCCGCCCATTGACGTCAATAATGACGTATGTTCCCATAGTAACGCCAATAGGGACTTTCCATTGACGTCAATGGGTGGAGTATTTACGGTAAACTGCCCACTTGGCAGTACATCAAGTGTATCATATGCCAAGTACGCCCCCTATTGACGTCAATGACGGTAAATGGCCCGCCTGGCATTATGCCCAGTACATGACCTTATGGGACTTTCCTACTTGGCAGTACATCTACGTATTAGTCATCGCTATTACCATGGTGATGCGGTTTTGGCAGTACATCAATGGGCGTGGATAGCGGTTTGACTCACGGGGATTTCCAAGTCTCCACCCCATTGACGTCAATGGGAGTTTGTTTTGGCACCAAAATCAACGGGACTTTCCAAAATGTCGTAACAACTCCGCCCCATTGACGCAAATGGGCGGTAGGCGTGTACGGTGGGAGGTCTATATAAGCAGAGCTGGTTTAGTGAACCGTCAGATCCGCTAGCGCTACCGGTCGCCACCATGGCTTCCCCCCCAAGGCACGGCCCCCCTGGACCTGCTTCTGGCGATGGACCTAATCTGAACAATAACAATAACAATAACAATCACTCCGTCCGGAAGTGCGGATACCTGAGAAAGCAGAAACATGGCCACAAACGATTCTTTGTGCTGCGAGGACCAGGAGCAGGAGGGGACGAGGCTACCGCAGGAGGAGGAAGCGCTCCTCAGCCACCTAGACTGGAATACTATGAAAGTGAGAAGAAATGGAGGTCAAAGGCAGGAGCTCCAAAACGCGTGATCGCACTGGACTGCTGTCTGAACATTAATAAGAGGGCAGATGCCAAGCACAAATACCTGATCGCCCTGTATACTAAAGATGAGTACTTTGCTGTCGCCGCTGAAAACGAGCAGGAACAGGAGGGCTGGTATCGAGCACTGACCGACCTGGTGTCCGAAGGACGAGCAGCAGCTGGAGATGCTCCACCAGCAGCAGCTCCAGCAGCATCATGCAGCGCATCCCTGCCAGGAGCTCTGGGAGGATCTGCAGGAGCTGCAGGAGCAGAGGACAGTTATGGACTGGTGGCTCCAGCAACAGCCGCTTACCGCGAAGTGTGGCAGGTCAACCTGAAGCCCAAAGGGCTGGGACAGTCTAAGAATCTGACCGGAGTGTACAGGCTGTGCCTGAGCGCACGCACAATCGGATTCGTGAAACTGAACTGTGAGCAGCCCAGCGTCACCCTGCAGCTGATGAATATCCGGAGATGTGGCCACTCTGATAGTTTCTTTTTCATTGAAGTGGGAAGATCCGCAGTCACAGGACCTGGGGAACTGTGGATGCAGGCTGACGATTCTGTGGTCGCACAGAACATCCATGAAACTATTCTGGAGGCCATGAAGGCTCTGAAAGAACTGTTTGAGTTCCGCCCCCGATCAAAGAGCCAGAGCTCCGGATCTAGTGCAACCCACCCTATTAGCGTGCCAGGAGCAAGGCGACACCATCACCTGGTCAATCTGCCTCCATCCCAGACAGGCCTGGTGCGACGGTCACGAACTGACAGCCTGGCAGCAACCCCACCTGCTGCAAAATGCTCAAGCTGTCGGGTGAGAACAGCCTCCGAGGGCGATGGGGGAGCAGCTGCAGGAGCAGCTGCAGCAGGAGCACGGCCTGTGTCTGTCGCTGGGAGTCCACTGTCACCAGGACCCGTGAGAGCCCCTCTGTCCAGGTCTCATACTCTGAGCGGAGGATGCGGAGGAAGAGGATCCAAGGTCGCACTGCTGCCAGCAGGAGGAGCACTGCAGCATAGTAGGTCAATGAGCATGCCAGTGGCACACAGCCCACCAGCTGCAACCAGCCCAGGATCCCTGTCCTCTAGTTCAGGACACGGCTCCGGATCTTATCCTCCACCACCTGGACCACATCCACCACTGCCTCACCCACTGCATCACGGACCAGGACAGAGGCCTAGCTCCGGAAGTGCATCAGCTAGCGGATCCCCATCTGATCCCGGCTTCATGTCCCTGGACGAGTACGGCTCTAGTCCTGGGGACCTGCGAGCCTTCTGCTCTCATCGGAGTAACACCCCAGAAAGCATCGCTGAGACACCTCCAGCAAGAGACGGAGGAGGAGGAGGAGAATTTTACGGCTATATGACAATGGATCGGCCACTGAGTCACTGTGGGAGATCATATAGAAGGGTGAGCGGAGACGCAGCTCAGGACCTGGATAGGGGCCTGAGGAAGCGCACTTACTCTCTGACCACACCAGCACGACAGCGACCAGTGCCTCAGCCATCAAGCGCTAGCCTGGATGAGTATACCCTGATGCGAGCAACATTCAGTGGCTCAGCCGGGAGGCTGTGCCCTTCCTGTCCAGCTTCCTCTCCCAAAGTGGCATACCATCCCTATCCTGAAGACTATGGAGATATCGAGATTGGCAGCCACCGGAGTTCAAGCTCCAATCTGGGAGCCGACGATGGCTACATGCCAATGACCCCTGGAGCAGCACTGGCTGGAAGCGGATCCGGCTCTTGTCGCAGTGACGATTACATGCCAATGTCACCCGCCAGTGTGTCAGCTCCCAAGCAGATCCTGCAGCCTCGAGCTGCAGCAGCTGCAGCAGCTGCAGTGCCATCCGCTGGACCAGCAGGACCAGCACCCACATCAGCCGCTGGCCGCACTTTTCCTGCCAGTGGAGGCGGGTATAAAGCATCTAGTCCTGCCGAATCAAGCCCAGAGGACAGCGGCTACATGCGGATGTGGTGCGGGTCAAAGCTGAGCATGGAGCATGCCGATGGCAAACTGCTGCCTAACGGGGACTACCTGAATGTGAGCCCATCCGATGCAGTCACTACCGGAACCCCACCTGACTTCTTCAGCGCAGCACTGCACCCTGGAGGAGAACCACTGCGAGGAGTGCCCGGATGCTGTTATTCCTCTCTGCCACGGAGCTACAAGGCCCCCTATACATGTGGGGGAGACTCCGATCAGTACGTGCTGATGAGTTCACCCGTCGGAAGAATTCTGGAGGAAGAGAGGCTGGAGCCTCAGGCAACTCCTGGACCATCCCAGGCTGCATCTGCTTTTGGGGCAGGACCTACCCAGCCACCACATCCAGTGGTCCCATCCCCTGTCCGCCCATCTGGAGGACGACCTGAGGGCTTCCTGGGACAGAGAGGAAGGGCCGTGAGGCCTACACGACTGAGCCTGGAGGGCCTGCCTTCCCTGCCATCTATGCACGAATATCCACTGCCTCCAGAGCCCAAGTCCCCTGGCGAATACATCAACATTGACTTTGGCGAGCCAGGAGCAAGACTGTCCCCACCTGCACCACCACTGCTGGCTTCTGCAGCTAGCTCCTCTAGTCTGCTGAGCGCTTCAAGCCCAGCATCCTCTCTGGGATCCGGAACACCTGGCACTAGTTCAGACTCTCGCCAGCGAAGTCCACTGTCAGATTATATGAATCTGGACTTCAGCTCCCCAAAGTCCCCAAAACCAGGAGCACCAAGCGGACACCCTGTGGGATCCCTGGATGGACTGCTGTCTCCAGAGGCCTCTAGTCCCTATCCTCCACTGCCCCCTAGACCATCTGCTAGTCCCTCAAGCTCCCTGCAGCCACCTCCTCCACCACCTGCACCAGGAGAACTGTACAGGCTGCCACCAGCAAGCGCAGTGGCTACCGCACAGGGACCTGGAGCAGCATCTAGTCTGTCAAGCGACACTGGAGATAACGGCGACTACACCGAGATGGCCTTTGGCGTGGCTGCAACTCCTCCACAGCCCATCGCCGCTCCCCCTAAGCCTGAAGCAGCAAGAGTGGCAAGCCCTACCTCCGGAGTCAAAAGGCTGAGTCTGATGGAGCAGGTGTCAGGGGTCGAAGCCTTCCTGCAGGCTTCCCAGCCACCAGACCCTCATCGAGGCGCAAAAGTGATCCGAGCAGATCCACAGGGAGGACGACGACGACACTCCTCTGAGACCTTCAGCTCAACAACTACCGTGACTCCAGTCTCACCCAGCTTCGCTCATAACCCCAAGAGACACAATTCCGCATCTGTGGAGAATGTCTCTCTGAGGAAAAGCTCCGAAGGAGGAGTGGGAGTCGGACCAGGAGGAGGAGACGAGCCTCCAACCAGCCCTCGACAGCTGCAGCCAGCACCCCCTCTGGCCCCCCAGGGACGGCCTTGGACACCAGGACAGCCAGGAGGACTGGTGGGATGCCCAGGATCTGGAGGAAGTCCTATGAGAAGGGAGACCAGCGCCGGATTCCAGAACGGCCTGAATTACATCGCTATTGACGTGCGGGAAGAGCCAGGACTGCCACCACAGCCACAGCCACCACCACCTCCACTGCCTCAGCCAGGGGATAAGTCTAGTTGGGGACGGACAAGAAGCCTGGGAGGACTGATCTCCGCAGTGGGAGTCGGATCTACTGGAGGAGGATGTGGAGGACCAGGACCTGGAGCTCTGCCCCCTGCAAATACCTACGCATCCATTGACTTTCTGTCCCATCATCTGAAGGAAGCAACTATCGTGAAGGAAGACTATAAAGACGACGACGACAAATAATCAGCCATACCACATTTGTAGAGGTTTTACTTGCTTTAAAAAACCTCCCACACCTCCCCCTGAACCTGAAACATAAAATGAATGCAATTGTTGTTGTTAACTTGTTTATTGCAGCTTATAATGGTTACAAATAAAGCAATAGCATCACAAATTTCACAAATAAAGCATTTTTTTCACTGCATTCTAGTTGTGGTTTGTCCAAACTCATCAATGTATCTTAACGCGTAAATTGTAAGCGTTAATATTTTGTTAAAATTCGCGTTAAATTTTTGTTAAATCAGCTCATTTTTTAACCAATAGGCCGAAATCGGCAAAATCCCTTATAAATCAAAAGAATAGACCGAGATAGGGTTGAGTGTTGTTCCAGTTTGGAACAAGAGTCCACTATTAAAGAACGTGGACTCCAACGTCAAAGGGCGAAAAACCGTCTATCAGGGCGATGGCCCACTACGTGAACCATCACCCTAATCAAGTTTTTTGGGGTCGAGGTGCCGTAAAGCACTAAATCGGAACCCTAAAGGGAGCCCCCGATTTAGAGCTTGACGGGGAAAGCCGGCGAACGTGGCGAGAAAGGAAGGGAAGAAAGCGAAAGGAGCGGGCGCTAGGGCGCTGGCAAGTGTAGCGGTCACGCTGCGCGTAACCACCACACCCGCCGCGCTTAATGCGCCGCTACAGGGCGCGTCAGGTGGCACTTTTCGGGGAAATGTGCGCGGAACCCCTATTTGTTTATTTTTCTAAATACATTCAAATATGTATCCGCTCATGAGACAATAACCCTGATAAATGCTTCAATAATATTGAAAAAGGAAGAGTCCTGAGGCGGAAAGAACCAGCTGTGGAATGTGTGTCAGTTAGGGTGTGGAAAGTCCCCAGGCTCCCCAGCAGGCAGAAGTATGCAAAGCATGCATCTCAATTAGTCAGCAACCAGGTGTGGAAAGTCCCCAGGCTCCCCAGCAGGCAGAAGTATGCAAAGCATGCATCTCAATTAGTCAGCAACCATAGTCCCGCCCCTAACTCCGCCCATCCCGCCCCTAACTCCGCCCAGTTCCGCCCATTCTCCGCCCCATGGCTGACTAATTTTTTTTATTTATGCAGAGGCCGAGGCCGCCTCGGCCTCTGAGCTATTCCAGAAGTAGTGAGGAGGCTTTTTTGGAGGCCTAGGCTTTTGCAAAGATCGATCAAGAGACAGGATGAGGATCGTTTCGCATGATTGAACAAGATGGATTGCACGCAGGTTCTCCGGCCGCTTGGGTGGAGAGGCTATTCGGCTATGACTGGGCACAACAGACAATCGGCTGCTCTGATGCCGCCGTGTTCCGGCTGTCAGCGCAGGGGCGCCCGGTTCTTTTTGTCAAGACCGACCTGTCCGGTGCCCTGAATGAACTGCAAGACGAGGCAGCGCGGCTATCGTGGCTGGCCACGACGGGCGTTCCTTGCGCAGCTGTGCTCGACGTTGTCACTGAAGCGGGAAGGGACTGGCTGCTATTGGGCGAAGTGCCGGGGCAGGATCTCCTGTCATCTCACCTTGCTCCTGCCGAGAAAGTATCCATCATGGCTGATGCAATGCGGCGGCTGCATACGCTTGATCCGGCTACCTGCCCATTCGACCACCAAGCGAAACATCGCATCGAGCGAGCACGTACTCGGATGGAAGCCGGTCTTGTCGATCAGGATGATCTGGACGAAGAGCATCAGGGGCTCGCGCCAGCCGAACTGTTCGCCAGGCTCAAGGCGAGCATGCCCGACGGCGAGGATCTCGTCGTGACCCATGGCGATGCCTGCTTGCCGAATATCATGGTGGAAAATGGCCGCTTTTCTGGATTCATCGACTGTGGCCGGCTGGGTGTGGCGGACCGCTATCAGGACATAGCGTTGGCTACCCGTGATATTGCTGAAGAGCTTGGCGGCGAATGGGCTGACCGCTTCCTCGTGCTTTACGGTATCGCCGCTCCCGATTCGCAGCGCATCGCCTTCTATCGCCTTCTTGACGAGTTCTTCTGAGCGGGACTCTGGGGTTCGAAATGACCGACCAAGCGACGCCCAACCTGCCATCACGAGATTTCGATTCCACCGCCGCCTTCTATGAAAGGTTGGGCTTCGGAATCGTTTTCCGGGACGCCGGCTGGATGATCCTCCAGCGCGGGGATCTCATGCTGGAGTTCTTCGCCCACCCTAGGGGGAGGCTAACTGAAACACGGAAGGAGACAATACCGGAAGGAACCCGCGCTATGACGGCAATAAAAAGACAGAATAAAACGCACGGTGTTGGGTCGTTTGTTCATAAACGCGGGGTTCGGTCCCAGGGCTGGCACTCTGTCGATACCCCACCGAGACCCCATTGGGGCCAATACGCCCGCGTTTCTTCCTTTTCCCCACCCCACCCCCCAAGTTCGGGTGAAGGCCCAGGGCTCGCAGCCAACGTCGGGGCGGCAGGCCCTGCCATAGCCTCAGGTTACTCATATATACTTTAGATTGATTTAAAACTTCATTTTTAATTTAAAAGGATCTAGGTGAAGATCCTTTTTGATAATCTCATGACCAAAATCCCTTAACGTGAGTTTTCGTTCCACTGAGCGTCAGACCCCGTAGAAAAGATCAAAGGATCTTCTTGAGATCCTTTTTTTCTGCGCGTAATCTGCTGCTTGCAAACAAAAAAACCACCGCTACCAGCGGTGGTTTGTTTGCCGGATCAAGAGCTACCAACTCTTTTTCCGAAGGTAACTGGCTTCAGCAGAGCGCAGATACCAAATACTGTCCTTCTAGTGTAGCCGTAGTTAGGCCACCACTTCAAGAACTCTGTAGCACCGCCTACATACCTCGCTCTGCTAATCCTGTTACCAGTGGCTGCTGCCAGTGGCGATAAGTCGTGTCTTACCGGGTTGGACTCAAGACGATAGTTACCGGATAAGGCGCAGCGGTCGGGCTGAACGGGGGGTTCGTGCACACAGCCCAGCTTGGAGCGAACGACCTACACCGAACTGAGATACCTACAGCGTGAGCTATGAGAAAGCGCCACGCTTCCCGAAGGGAGAAAGGCGGACAGGTATCCGGTAAGCGGCAGGGTCGGAACAGGAGAGCGCACGAGGGAGCTTCCAGGGGGAAACGCCTGGTATCTTTATAGTCCTGTCGGGTTTCGCCACCTCTGACTTGAGCGTCGATTTTTGTGATGCTCGTCAGGGGGGCGGAGCCTATGGAAAAACGCCAGCAACGCGGCCTTTTTACGGTTCCTGGCCTTTTGCTGGCCTTTTGCTCACATGTTCTTTCCTGCGTTATCCCCTGATTCTGTGGATAACCGTATTACCGCCATGCAT |
| PDPK1-TagRFP-T | TAGTTATTAATAGTAATCAATTACGGGGTCATTAGTTCATAGCCCATATATGGAGTTCCGCGTTACATAACTTACGGTAAATGGCCCGCCTGGCTGACCGCCCAACGACCCCCGCCCATTGACGTCAATAATGACGTATGTTCCCATAGTAACGCCAATAGGGACTTTCCATTGACGTCAATGGGTGGAGTATTTACGGTAAACTGCCCACTTGGCAGTACATCAAGTGTATCATATGCCAAGTACGCCCCCTATTGACGTCAATGACGGTAAATGGCCCGCCTGGCATTATGCCCAGTACATGACCTTATGGGACTTTCCTACTTGGCAGTACATCTACGTATTAGTCATCGCTATTACCATGGTGATGCGGTTTTGGCAGTACATCAATGGGCGTGGATAGCGGTTTGACTCACGGGGATTTCCAAGTCTCCACCCCATTGACGTCAATGGGAGTTTGTTTTGGCACCAAAATCAACGGGACTTTCCAAAATGTCGTAACAACTCCGCCCCATTGACGCAAATGGGCGGTAGGCGTGTACGGTGGGAGGTCTATATAAGCAGAGCTGGTTTAGTGAACCGTCAGATCCGCTAGCGCTACCGGTCGCCACCATGGCCAGGACCACCAGCCAGCTGTATGACGCCGTGCCCATCCAGTCCAGCGTGGTGTTATGTTCCTGCCCATCCCCATCAATGGTGAGGACCCAGACTGAGTCCAGCACGCCCCCTGGCATTCCTGGTGGCAGCAGGCAGGGCCCCGCCATGGACGGCACTGCAGCCGAGCCTCGGCCCGGCGCCGGCTCCCTGCAGCATGCCCAGCCTCCGCCGCAGCCTCGGAAGAAGCGGCCTGAGGACTTCAAGTTTGGGAAAATCCTTGGGGAAGGCTCTTTTTCCACGGTTGTCCTGGCTCGAGAACTGGCAACCTCCAGAGAATATGCGATTAAAATTCTGGAGAAGCGACATATCATAAAAGAGAACAAGGTCCCCTATGTAACCAGAGAGCGGGATGTCATGTCGCGCCTGGATCACCCCTTCTTTGTTAAGCTTTACTTCACATTTCAGGACGACGAGAAGCTGTATTTCGGCCTTAGTTATGCCAAAAATGGAGAACTACTTAAATATATTCGCAAAATCGGTTCATTCGATGAGACCTGTACCCGATTTTACACGGCTGAGATTGTGTCTGCTTTAGAGTACTTGCACGGCAAGGGCATCATTCACAGGGACCTTAAACCGGAAAACATTTTGTTAAATGAAGATATGCACATCCAGATCACAGATTTTGGAACAGCAAAAGTCTTATCCCCAGAGAGCAAACAAGCCAGGGCCAACTCATTCGTGGGAACAGCGCAGTACGTTTCTCCAGAGCTGCTCACGGAGAAGTCCGCCTGTAAGAGTTCAGACCTTTGGGCTCTTGGATGCATAATATACCAGCTTGTGGCAGGACTCCCACCATTCCGAGCTGGAAACGAGTATCTTATATTTCAGAAGATCATTAAGTTGGAATATGACTTTCCAGAAAAATTCTTCCCTAAGGCAAGAGACCTCGTGGAGAAACTTTTGGTTTTAGATGCCACAAAGCGGTTAGGCTGTGAGGAAATGGAAGGATACGGACCTCTTAAAGCACACCCGTTCTTCGAGTCCGTCACGTGGGAGAACCTGCACCAGCAGACGCCTCCGAAGCTCACCGCTTACCTGCCGGCTATGTCGGAAGACGACGAGGACTGCTATGGCAATTATGACAATCTCCTGAGCCAGTTTGGCTGCATGCAGGTGTCTTCGTCCTCCTCCTCACACTCCCTGTCAGCCTCCGACACGGGCCTGCCCCAGAGGTCAGGCAGCAACATAGAGCAGTACATTCACGATCTGGACTCGAACTCCTTTGAACTGGACTTACAGTTTTCCGAAGATGAGAAGAGGTTGTTGTTGGAGAAGCAGGCTGGCGGAAACCCTTGGCACCAGTTTGTAGAAAATAATTTAATACTAAAGATGGGCCCAGTGGATAAGCGGAAGGGTTTATTTGCAAGACGACGACAGCTGTTGCTCACAGAAGGACCACATTTATATTATGTGGATCCTGTCAACAAAGTTCTGAAAGGTGAAATTCCTTGGTCACAAGAACTTCGACCAGAGGCCAAGAATTTTAAAACTTTCTTTGTCCACACGCCTAACAGGACGTATTATCTGATGGACCCCAGCGGGAACGCACACAAGTGGTGCAGGAAGATCCAGGAGGTTTGGAGGCAGCGATACCAGAGCCACCCGGACGCCGCTGTGCAGGTGTCTAAGGGCGAAGAGCTGATTAAGGAGAACATGCACATGAAGCTGTACATGGAGGGCACCGTGAACAACCACCACTTCAAGTGCACATCCGAGGGCGAAGGCAAGCCCTACGAGGGCACCCAGACCATGAGAATCAAGGTGGTCGAGGGCGGCCCTCTCCCCTTCGCCTTCGACATCCTGGCTACCAGCTTCATGTACGGCAGCAGAACCTTCATCAACCACACCCAGGGCATCCCCGATTTCTTTAAGCAGTCCTTCCCTGAGGGCTTCACATGGGAGAGAGTCACCACATACGAAGACGGGGGCGTGCTGACCGCTACCCAGGACACCAGCCTCCAGGACGGCTGCCTCATCTACAACGTCAAGATCAGAGGGGTGAACTTCCCATCCAACGGCCCTGTGATGCAGAAGAAAACACTCGGCTGGGAGGCCAACACCGAGATGCTGTACCCCGCTGACGGCGGCCTGGAAGGCAGAACCGACATGGCCCTGAAGCTCGTGGGCGGGGGCCACCTGATCTGCAACTTCAAGACCACATACAGATCCAAGAAACCCGCTAAGAACCTCAAGATGCCCGGCGTCTACTATGTGGACCACAGACTGGAAAGAATCAAGGAGGCCGACAAAGAGACCTACGTCGAGCAGCACGAGGTGGCTGTGGCCAGATACTGCGACCTCCCTAGCAAACTGGGGCACAAACTTAATGGCATGGACGAGCTGTACAAGTGAAATCAGCCATACCACATTTGTAGAGGTTTTACTTGCTTTAAAAAACCTCCCACACCTCCCCCTGAACCTGAAACATAAAATGAATGCAATTGTTGTTGTTAACTTGTTTATTGCAGCTTATAATGGTTACAAATAAAGCAATAGCATCACAAATTTCACAAATAAAGCATTTTTTTCACTGCATTCTAGTTGTGGTTTGTCCAAACTCATCAATGTATCTTAACGCGTAAATTGTAAGCGTTAATATTTTGTTAAAATTCGCGTTAAATTTTTGTTAAATCAGCTCATTTTTTAACCAATAGGCCGAAATCGGCAAAATCCCTTATAAATCAAAAGAATAGACCGAGATAGGGTTGAGTGTTGTTCCAGTTTGGAACAAGAGTCCACTATTAAAGAACGTGGACTCCAACGTCAAAGGGCGAAAAACCGTCTATCAGGGCGATGGCCCACTACGTGAACCATCACCCTAATCAAGTTTTTTGGGGTCGAGGTGCCGTAAAGCACTAAATCGGAACCCTAAAGGGAGCCCCCGATTTAGAGCTTGACGGGGAAAGCCGGCGAACGTGGCGAGAAAGGAAGGGAAGAAAGCGAAAGGAGCGGGCGCTAGGGCGCTGGCAAGTGTAGCGGTCACGCTGCGCGTAACCACCACACCCGCCGCGCTTAATGCGCCGCTACAGGGCGCGTCAGGTGGCACTTTTCGGGGAAATGTGCGCGGAACCCCTATTTGTTTATTTTTCTAAATACATTCAAATATGTATCCGCTCATGAGACAATAACCCTGATAAATGCTTCAATAATATTGAAAAAGGAAGAGTCCTGAGGCGGAAAGAACCAGCTGTGGAATGTGTGTCAGTTAGGGTGTGGAAAGTCCCCAGGCTCCCCAGCAGGCAGAAGTATGCAAAGCATGCATCTCAATTAGTCAGCAACCAGGTGTGGAAAGTCCCCAGGCTCCCCAGCAGGCAGAAGTATGCAAAGCATGCATCTCAATTAGTCAGCAACCATAGTCCCGCCCCTAACTCCGCCCATCCCGCCCCTAACTCCGCCCAGTTCCGCCCATTCTCCGCCCCATGGCTGACTAATTTTTTTTATTTATGCAGAGGCCGAGGCCGCCTCGGCCTCTGAGCTATTCCAGAAGTAGTGAGGAGGCTTTTTTGGAGGCCTAGGCTTTTGCAAAGATCGATCAAGAGACAGGATGAGGATCGTTTCGCATGATTGAACAAGATGGATTGCACGCAGGTTCTCCGGCCGCTTGGGTGGAGAGGCTATTCGGCTATGACTGGGCACAACAGACAATCGGCTGCTCTGATGCCGCCGTGTTCCGGCTGTCAGCGCAGGGGCGCCCGGTTCTTTTTGTCAAGACCGACCTGTCCGGTGCCCTGAATGAACTGCAAGACGAGGCAGCGCGGCTATCGTGGCTGGCCACGACGGGCGTTCCTTGCGCAGCTGTGCTCGACGTTGTCACTGAAGCGGGAAGGGACTGGCTGCTATTGGGCGAAGTGCCGGGGCAGGATCTCCTGTCATCTCACCTTGCTCCTGCCGAGAAAGTATCCATCATGGCTGATGCAATGCGGCGGCTGCATACGCTTGATCCGGCTACCTGCCCATTCGACCACCAAGCGAAACATCGCATCGAGCGAGCACGTACTCGGATGGAAGCCGGTCTTGTCGATCAGGATGATCTGGACGAAGAGCATCAGGGGCTCGCGCCAGCCGAACTGTTCGCCAGGCTCAAGGCGAGCATGCCCGACGGCGAGGATCTCGTCGTGACCCATGGCGATGCCTGCTTGCCGAATATCATGGTGGAAAATGGCCGCTTTTCTGGATTCATCGACTGTGGCCGGCTGGGTGTGGCGGACCGCTATCAGGACATAGCGTTGGCTACCCGTGATATTGCTGAAGAGCTTGGCGGCGAATGGGCTGACCGCTTCCTCGTGCTTTACGGTATCGCCGCTCCCGATTCGCAGCGCATCGCCTTCTATCGCCTTCTTGACGAGTTCTTCTGAGCGGGACTCTGGGGTTCGAAATGACCGACCAAGCGACGCCCAACCTGCCATCACGAGATTTCGATTCCACCGCCGCCTTCTATGAAAGGTTGGGCTTCGGAATCGTTTTCCGGGACGCCGGCTGGATGATCCTCCAGCGCGGGGATCTCATGCTGGAGTTCTTCGCCCACCCTAGGGGGAGGCTAACTGAAACACGGAAGGAGACAATACCGGAAGGAACCCGCGCTATGACGGCAATAAAAAGACAGAATAAAACGCACGGTGTTGGGTCGTTTGTTCATAAACGCGGGGTTCGGTCCCAGGGCTGGCACTCTGTCGATACCCCACCGAGACCCCATTGGGGCCAATACGCCCGCGTTTCTTCCTTTTCCCCACCCCACCCCCCAAGTTCGGGTGAAGGCCCAGGGCTCGCAGCCAACGTCGGGGCGGCAGGCCCTGCCATAGCCTCAGGTTACTCATATATACTTTAGATTGATTTAAAACTTCATTTTTAATTTAAAAGGATCTAGGTGAAGATCCTTTTTGATAATCTCATGACCAAAATCCCTTAACGTGAGTTTTCGTTCCACTGAGCGTCAGACCCCGTAGAAAAGATCAAAGGATCTTCTTGAGATCCTTTTTTTCTGCGCGTAATCTGCTGCTTGCAAACAAAAAAACCACCGCTACCAGCGGTGGTTTGTTTGCCGGATCAAGAGCTACCAACTCTTTTTCCGAAGGTAACTGGCTTCAGCAGAGCGCAGATACCAAATACTGTCCTTCTAGTGTAGCCGTAGTTAGGCCACCACTTCAAGAACTCTGTAGCACCGCCTACATACCTCGCTCTGCTAATCCTGTTACCAGTGGCTGCTGCCAGTGGCGATAAGTCGTGTCTTACCGGGTTGGACTCAAGACGATAGTTACCGGATAAGGCGCAGCGGTCGGGCTGAACGGGGGGTTCGTGCACACAGCCCAGCTTGGAGCGAACGACCTACACCGAACTGAGATACCTACAGCGTGAGCTATGAGAAAGCGCCACGCTTCCCGAAGGGAGAAAGGCGGACAGGTATCCGGTAAGCGGCAGGGTCGGAACAGGAGAGCGCACGAGGGAGCTTCCAGGGGGAAACGCCTGGTATCTTTATAGTCCTGTCGGGTTTCGCCACCTCTGACTTGAGCGTCGATTTTTGTGATGCTCGTCAGGGGGGCGGAGCCTATGGAAAAACGCCAGCAACGCGGCCTTTTTACGGTTCCTGGCCTTTTGCTGGCCTTTTGCTCACATGTTCTTTCCTGCGTTATCCCCTGATTCTGTGGATAACCGTATTACCGCCATGCAT |

**Supplementary File 3b.** DNA sequence of primers used to generate plasmids in this study.

| **Plasmid** | **Cloning Method** | **Primer DNA Sequence (5'-3')** |
| --- | --- | --- |
| PDPK1-eGFP | Gibson Assembly | ATGGCCAGGACCACCAGCCAGCTG |
|  |  | CTGCACAGCGGCGTCCGGGTGGCTCT |
|  |  | CAGCTGGCTGGTGGTCCTGGCCATGGTGGCGACCGGTAGCGCTAG |
|  |  | AGAGCCACCCGGACGCCGCTGTGCAGGTGAGCAAGGGCGAGGAGCTGTTCAC |
| IRS1-eGFP | Gibson Assembly | CTAGCGCTACCGGTCGCCACCATGGCGAGCCCTCCGGAGA |
|  |  | GTGAACAGCTCCTCGCCCTTGCTCACCTGACGGTCCTCTGGCTGCTTCTGG |
|  |  | GGTGGCGACCGGTAGCGCTAG |
|  |  | GTGAGCAAGGGCGAGGAGCTGTTCAC |
| IRS2-eGFP | Gibson Assembly | CTAGCGCTACCGGTCGCCACCATGGCTTCCCCCCCAAGGCA |
|  |  | GTGAACAGCTCCTCGCCCTTGCTCACTTCCTTCACGATAGTTGCTTCCTTCAG |
|  |  | GGTGGCGACCGGTAGCGCTAG |
|  |  | GTGAGCAAGGGCGAGGAGCTGTTCAC |
| IRS2-FLAG | Gibson Assembly | TTATTTGTCGTCGTCGTCTTTATAGTCTTCCTTCACGATAGTTGCTTCC |
|  |  | GACTATAAAGACGACGACGACAAATAATCAGCCATACCACATTTGTAG |
|  |  | CAGGATGATCTGGACGAAGAGC |
|  |  | GCTCTTCGTCCAGATCATCCTG |
| PDPK1-TagRFP-T | Gibson Assembly | AGAGCCACCCGGACGCCGCTGTGCAGGTGTCTAAGGGCGAAGAGCTG |
|  |  | CCTCTACAAATGTGGTATGGCTGATTTCACTTGTACAGCTCGTCCATGCCA |
|  |  | AATCAGCCATACCACATTTGTAGAGG |
|  |  | CTGCACAGCGGCGTCCGGGTGGCTCT |
| IRS1-eGFP DelPH | Gibson Assembly | CATGGTGGCGACCGGTAGCGCTAG |
|  |  | CTAGCGCTACCGGTCGCCACCATGCGTGCTAAGGGCCACCACGAC |
|  |  | CAGGATGATCTGGACGAAGAGC |
|  |  | GCTCTTCGTCCAGATCATCCTG |
| IRS1-eGFP DelPTB | Gibson Assembly | ACGAGGACTGGCTCTTGCTGCGAGGGAATGCGGGTCCTGGGGGCACGTCA |
|  |  | CCTCGCAGCAAGAGCCAGTCCTCGT |
|  |  | CAGGATGATCTGGACGAAGAGC |
|  |  | GCTCTTCGTCCAGATCATCCTG |
| TagRFP-T-Akt2 W80A | Site Directed Mutagenesis | CATACGCTGCCTGCAGGCGACCACAGTCATCGAGAGG |
| TagRFP-T-Akt2 W80A-T309A | Site Directed Mutagenesis | CATACGCTGCCTGCAGGCGACCACAGTCATCGAGAGG |
|  |  | GCATCAGTGACGGGGCCACCATGAAAGCCTTCTGTGGGACC |
| TagRFP-T-Akt2 W80A-S474A | Site Directed Mutagenesis | CATACGCTGCCTGCAGGCGACCACAGTCATCGAGAGG |
|  |  | ACTCGCGGATGCTGGCCGAGTAGGCGAACTGGGGGAAGTGGGTCCGCTGGTCCAGCTC |
| TagRFP-T-Akt2 W80A-T309A-S474A | Site Directed Mutagenesis | CATACGCTGCCTGCAGGCGACCACAGTCATCGAGAGG |
|  |  | GCATCAGTGACGGGGCCACCATGAAAGCCTTCTGTGGGACC |
|  |  | ACTCGCGGATGCTGGCCGAGTAGGCGAACTGGGGGAAGTGGGTCCGCTGGTCCAGCTC |
| IRS1-eGFP 6P | Site Directed Mutagenesis | GAGTGATGAGTTCCGCCCTCGCAGCAAGGCCCAGTCCTCGTCCAACTGCTC |
|  |  | CTGACCCGCCGATCACGCACTGAGGCCATCACCGCCACCTCCCCGGCCAGCA |
|  |  | GGACATGGTGCCTTCGCCGTCAGCGGAGGCGCGGACACGGAAGGAGCCTGGCT |
|  |  | GGTAATGGTAGGGGATGTGCCTGCCGCGTGAGCTCTCTTTCGGAACCGATTATCCAG |
|  |  | CACTGGGTGTTGAGGAGAAAGTCTCGGCGCTATGCCTCCGCCGGCACCCTTGTG |
| IRS1-eGFP S270A | Site Directed Mutagenesis | GAGTGATGAGTTCCGCCCTCGCAGCAAGGCCCAGTCCTCGTCCAACTGCTC |
| IRS1-eGFP S307A | Site Directed Mutagenesis | CTGACCCGCCGATCACGCACTGAGGCCATCACCGCCACCTCCCCGGCCAGCA |
| IRS1-eGFP S330A | Site Directed Mutagenesis | GGACATGGTGCCTTCGCCGTCAGCGGAGGCGCGGACACGGAAGGAGCCTGGCT |
| IRS1-eGFP T525A | Site Directed Mutagenesis | GGTAATGGTAGGGGATGTGCCTGCCGAGTGAGCTCTCTTTCGGAACCGATTATCCAG |
| IRS1-eGFP S527A | Site Directed Mutagenesis | GGTAATGGTAGGGGATGTGCCTGCCGCGTGAGTTCTCTTTCGGAACCGATTATCCAG |
| IRS1-eGFP S1101A | Site Directed Mutagenesis | CACTGGGTGTTGAGGAGAAAGTCTCGGCGCTATGCCTCCGCCGGCACCCTTGTG |
| IRS1-eGFP S270/527A | Site Directed Mutagenesis | GAGTGATGAGTTCCGCCCTCGCAGCAAGGCCCAGTCCTCGTCCAACTGCTC |
|  |  | GGTAATGGTAGGGGATGTGCCTGCCGCGTGAGTTCTCTTTCGGAACCGATTATCCAG |
| IRS2-eGFP 5P | Site Directed Mutagenesis | CTGTTTGAGTTCCGCCCCCGATCAAAGGCCCAGAGCTCCGGATCTAGTGCAACC |
|  |  | CTGGTGCGACGGTCACGAACTGACGCCCTGGCAGCAACCCCACCTGCTGCAAAATGC |
|  |  | CAGCTGCTCCCCCATCGCCCTCGGCGGCTGTTCTCACCCGACAGCTTG |
|  |  | CTGTCGTGCTGGTGTGGTCAGAGCGTAAGTGCGCTTCCTCAGGCCCCTATC |
|  |  | GGTAGTTGTTGAGCTGAAGGTCTCAGCGGAGTGTCGTCGTCGTCCTCCCTGTGGA |
| IRS2-eGFP S306A | Site Directed Mutagenesis | CTGTTTGAGTTCCGCCCCCGATCAAAGGCCCAGAGCTCCGGATCTAGTGCAACC |
| IRS2-eGFP S346A | Site Directed Mutagenesis | CTGGTGCGACGGTCACGAACTGACGCCCTGGCAGCAACCCCACCTGCTGCAAAATGC |
| IRS2-eGFP S365A | Site Directed Mutagenesis | CAGCTGCTCCCCCATCGCCCTCGGCGGCTGTTCTCACCCGACAGCTTG |
| IRS2-eGFP S577A | Site Directed Mutagenesis | CTGTCGTGCTGGTGTGGTCAGAGCGTAAGTGCGCTTCCTCAGGCCCCTATC |
| IRS2-eGFP S1149A | Site Directed Mutagenesis | GGTAGTTGTTGAGCTGAAGGTCTCAGCGGAGTGTCGTCGTCGTCCTCCCTGTGGA |
| IRS2-eGFP S306/577A | Site Directed Mutagenesis | CTGTTTGAGTTCCGCCCCCGATCAAAGGCCCAGAGCTCCGGATCTAGTGCAACC |
|  |  | CTGTCGTGCTGGTGTGGTCAGAGCGTAAGTGCGCTTCCTCAGGCCCCTATC |
